# Amplification of Photochemical Chiroptical Activity of Chiral Gold Nanocubes

**DOI:** 10.1002/smll.202505093

**Published:** 2025-06-22

**Authors:** Shashank K. Gahlaut, Oscar Avalos‐Ovando, Ryeong Myeong Kim, Ridwan Hussein, Sabrina Juergensen, Stephanie Reich, Alexander O. Govorov, Ki Tae Nam, Ilko Bald

**Affiliations:** ^1^ Institute of Chemistry University of Potsdam 14476 Potsdam Germany; ^2^ Department of Physics and Astronomy Nanoscale and Quantum Phenomena Institute Ohio University Athens OH 45701 USA; ^3^ Department of Nanoscience Joint School of Nanoscience & Nanoengineering University of North Carolina at Greensboro Greensboro NC 27401 USA; ^4^ Department of Materials Science and Engineering Seoul National University Seoul 08826 Republic of Korea; ^5^ Department of Physics Freie University Berlin 14195 Berlin Germany; ^6^ Dynamics of Molecules and Clusters Department J. Heyrovský Institute of Physical Chemistry of the CAS Dolejškova 3 Prague 18223 Czech Republic

**Keywords:** Au helicoids, chiral plasmonics, circular dichroism, dehalogenation, hot electrons, plasmonic chemistry, SERS

## Abstract

Chiral plasmonic nanostructures enable exceptionally high dissymmetry factors (g‐factors) compared to chiral molecules and present unparalleled opportunities in light manipulation, polarization‐sensitive photochemistry, and chiral sensing. Here polarization‐dependent plasmonic chemistry on chiral gold nanocubes (AuNCs) is presented, leveraging the high sensitivity of surface‐enhanced Raman scattering (SERS). The AuNCs exhibit strong optical activity and localized surface plasmon resonances acting as highly efficient nanoscale light antennae. Employing the hot electron‐induced dehalogenation of 8‐Bromoadenine as a model reaction, it is demonstrated that circularly polarized light induces asymmetric reaction rates due to circular dichroism (CD) in hot electron generation efficiency. Astonishingly, the photochemical g‐factor, quantified by the differential reaction rate coefficients under left‐handed and right‐handed circularly polarized light, surpasses its optical counterpart and can be further enhanced by laser intensity. Remarkably, multilayer assemblies of AuNCs exhibit a reversal in photochemical CD, which is tuneable via laser power and enables further g‐factor enhancement. Comprehensive electromagnetic simulations of extinction spectra and hot electron generation maps corroborate the profound impact of particle arrangement on the optical g‐factor and the g‐factor for hot‐electron generation. This work demonstrates a systematic approach to enhance the photochemical chiroptical response of chiral AuNCs, paving the way for extraordinary control over chemical reactions with light.

## Graphical abstract

“Chiral gold nanocubes (AuNCs) enable polarization‐dependent plasmonic catalysis with exceptional photochemical circular dichroism. Circularly polarized light drives hot‐electron‐induced reactions with asymmetric reaction rates, monitored via operando SERS, with photochemical g‐factors exceeding optical limits. Multilayer assemblies reveal reversible and tunable chiroptical responses. This work offers a strategy to amplify chiral‐selective photochemistry via nanostructure design and light control”.

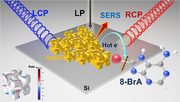

## 1 Introduction

Chiral plasmonics offers a powerful avenue for manipulating light–matter interactions by integrating structural chirality with localized surface plasmon resonances (LSPRs). Unlike natural biomolecules—such as proteins and DNA—which exhibit weak optical activity confined to the ultraviolet region, artificially engineered chiral plasmonic nanostructures can achieve dissymmetry factors (g‐factors) orders of magnitude higher, and critically, within the visible spectrum.^[^ ^1^ ^]^ This enhancement arises from the intense near fields and multipolar plasmonic modes supported by these nanostructures, enabling new applications in polarization‐sensitive photodetection, enantioselective sensing, and asymmetric photocatalysis. A central challenge in this field is the fabrication of colloidal chiral plasmonic nanostructures with well‐defined morphology and robust optical activity. Various nano‐fabrication tools like lithography,^[^ ^2^, ^3^, ^4^ ^]^ physical vapor deposition^[^ ^5^, ^6^, ^7^, ^8^, ^9^, ^10^ ^]^ colloidal synthesis^[^ ^11^, ^12^, ^13^, ^14^, ^15^, ^16^ ^]^ and DNA nanotechnology^[^ ^17^, ^18^, ^19^ ^]^ have been employed for manufacturing chiral nanostructures. Among recent advances, colloidal methods allow the transfer of molecular chirality to metallic nanoparticles through ligand‐mediated growth, often yielding high‐Miller‐index surfaces and helicoidal morphologies. In this work, we employ chiral gold nanocubes (AuNCs) of the 432 helicoid III type, which exhibit strong optical activity with a Kuhn's g‐factor of 0.18 due to their higher‐order plasmonic modes and large‐scale chiral features.^[^ ^11^, ^20^, ^21^ ^]^

Plasmon‐assisted photocatalysis exploits nonradiative LSPR decay to generate energetic charge carriers—hot electrons and holes—that can transfer into nearby molecules to trigger chemical reactions.^[^ ^22^, ^23^ ^]^ This mechanism offers nanometric spatial control and is tunable via structural parameters and excitation conditions such as wavelength, intensity, and polarization.^[^ ^24^ ^]^ While the concept of chiral photocatalysis using circularly polarized light (CPL) is theoretically established,^[^ ^25^ ^]^ direct experimental evidence linking optical chirality to asymmetric reaction kinetics remains sparse.^[^ ^26^, ^27^ ^]^ Furthermore, the role of nanoparticle assembly and environmental conditions in modulating photochemical chiral response is poorly understood.

Previous studies have explored how hot electron dynamics are influenced by chiral plasmonic geometries. For instance, the rate of hot electron generation from chiral hotspots has been both theoretically calculated and experimentally validated via photocurrent measurements in chiral Au and Ag arrays illuminated with left‐ and right‐handed circularly polarized light (LCP and RCP, respectively).^[^ ^15^, ^28^ ^]^ In another report, asymmetric photothermal heating was demonstrated for a single chiral helicoid under CPL excitation at a specific wavelength, revealing measurable temperature differences between LCP and RCP due to polarization‐dependent absorption cross‐sections and LSPR characteristics.^[^ ^29^ ^]^ Furthermore, geometric chirality in Au helicoids integrated with C₃N₄ nanosheets has been exploited to enhance photocatalytic hydrogen evolution by promoting charge separation and chiral‐induced spin selectivity at the metal–semiconductor interface.^[^ ^30^ ^]^ A recent study reports the inversion of chiroptical signals in individual plasmonic helicoids upon their deposition on metallic Au substrates.^[^ ^31^ ^]^ This effect was attributed to strong electromagnetic coupling between the helicoidal nanostructures and the reflective gold film, which altered the local field distribution and modified the sign of the circular dichroism (CD) signal. These findings collectively highlight the vast potential of chiral plasmonic systems to govern asymmetric light–matter interactions and energy conversion pathways.

Building on this foundation, the present study delves into hot‐electron driven plasmonic chemistry mediated by chiral light and explores the potential to boost and reconfigure the chiroptical response of pristine colloidal AuNCs through tunable illumination conditions and nanocube arrangement.

In this work, we bridge this gap by demonstrating polarization‐sensitive plasmonic chemistry on colloidal chiral AuNCs, using the hot‐electron‐driven dehalogenation of 8‐bromoadenine (BrA) as a model reaction.^[^ ^32^ ^]^ Hot electron transfer from the metal to the LUMO of the adsorbed BrA results in the formation of a transient negative ion, which then dissociates the C‐Br bond, a process referred to as dissociative electron attachment (DEA):^[^ ^32^, ^33^, ^34^, ^35^ ^]^

(1) $$AuNC+h\nu \to e^{-}+h^{+}$$

(2) $$BrA+e^{-}\to \;BrA^{*-}\to A^{\cdot }+Br^{-}$$

This reaction is tracked in situ via surface‐enhanced Raman scattering (SERS), which enables direct monitoring of reactant and product vibrational signatures over time. The ring breathing modes of the adduct (BrA at 770 cm^−1^) and product (Adenine A at 735 cm^−1^) are readily detectable by SERS. This makes SERS a powerful tool for monitoring these reactions through time‐dependent measurements and quantifying their reaction rates.^[^ ^36^, ^37^ ^]^

Our findings reveal a clear photochemical circular dichroism: reaction rates differ significantly under left‐ and right‐handed CPL, and the extracted photochemical g‐factor exceeds the optical g‐factor by a factor of two. This enhancement confirms that the chiral plasmonic response is not limited to light absorption but actively governs chemical reactivity. Most notably, we have discovered that multilayer assembly of AuNCs leads to a reversal in photochemical CD, a phenomenon that is strongly dependent on laser power. This provides an accessible and reversible strategy to amplify or tune reaction asymmetry simply by adjusting light intensity—a novel degree of control absent in molecular or achiral plasmonic systems.

Electromagnetic simulations further corroborate our experimental results, showing that both the arrangement of nanocubes and polarization of light profoundly influence hot electron generation maps and extinction spectra. The findings position chiral AuNCs as a versatile platform for tailoring asymmetric photocatalysis with tunable selectivity, enabling new approaches for CPL‐driven chemical transformations, spin‐selective processes, and optically programmable catalysis at the nanoscale.

## 2 Results and Discussion

**Figure 1:**
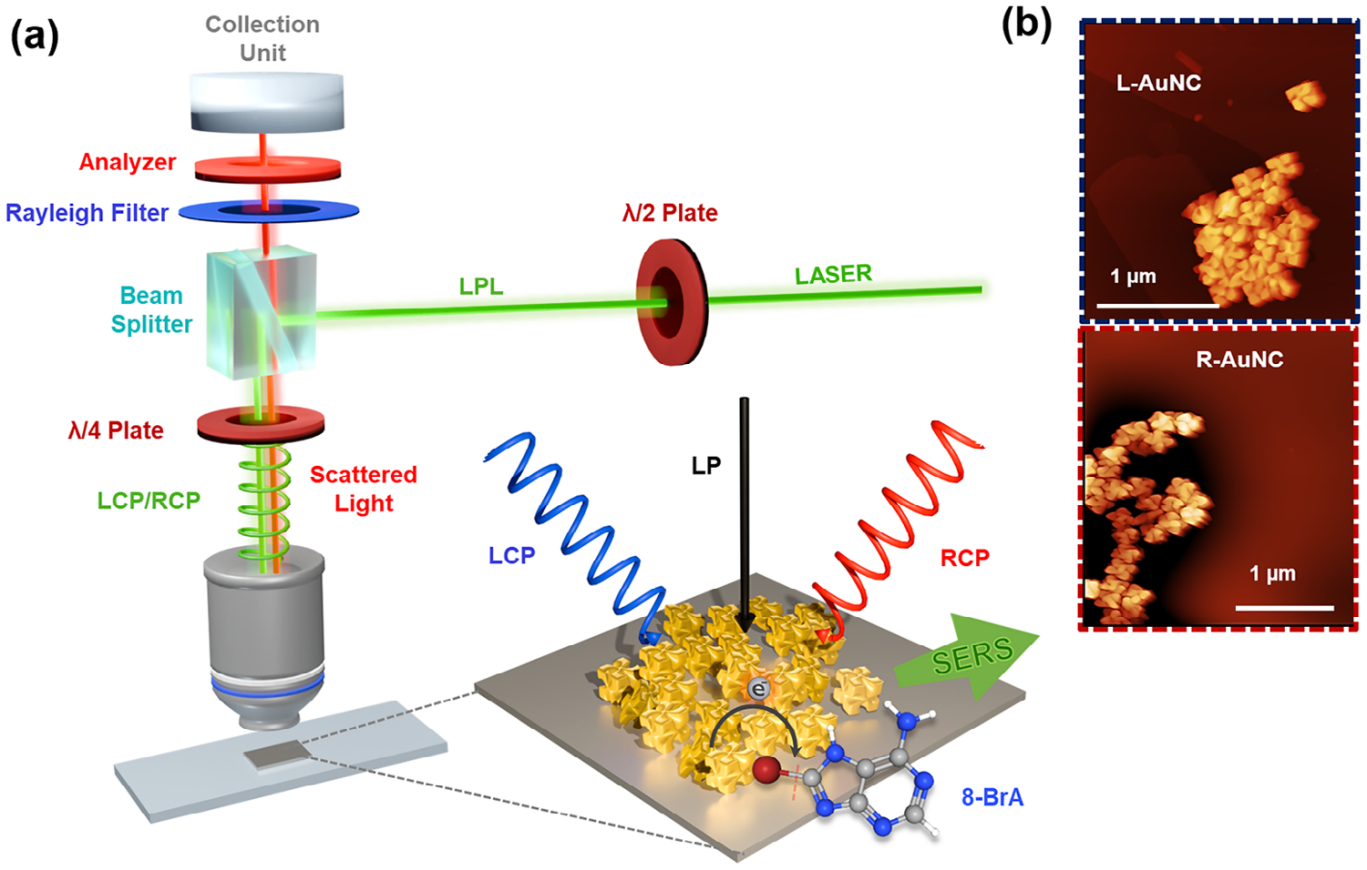
a) Schematic illustration of the CPL assisted SERS experiment with L‐AuNCs incubated with BrA molecules and dried on Si substrate, b) representative atomic force micrographs of L‐ and R‐AuNCs.

**Figure** **1**a illustrates the schematic of the experimental polarized‐Raman set‐up; showing chiral AuNC clusters with adsorbed BrA molecules dropcasted and dried on a cleaned Si substrate. The morphology and handedness of the synthesized left‐handed (L‐) and right‐handed (R‐) AuNCs were characterized by atomic force microscopy (AFM) (Figure 1b; Figure S1, Supporting Information), scanning electron microscopy (SEM) (Figure S2, Supporting Information), transmission electron microscopy (TEM), and dynamic light scattering (DLS) analysis (Figure S3, Supporting Information). Throughout this manuscript, blue and red color schemes are used to represent L‐ and R‐AuNCs, respectively. The synthesized AuNCs are uniform in size and shape with an average length of 180 nm as confirmed by the AFM height profile shown in Figure S1 (Supporting Information). They show strong optical activity in suspension with a g‐factor of 0.18, indicative of their robust chiral plasmonic response.

Time‐series SERS spectra were recorded under excitation with LCP, RCP and linearly polarized (LP) continuous‐wave (cw) laser beams. Although the sharp edges of individual AuNCs provide strong local electric field enhancement sufficient for single‐particle SERS, the resulting signal was insufficient for kinetic time‐trace analysis. Therefore, measurements were conducted on dried ensembles of AuNCs, leveraging interparticle hotspots within aggregates to amplify the SERS signal for kinetic evaluation.

**Figure 2:**
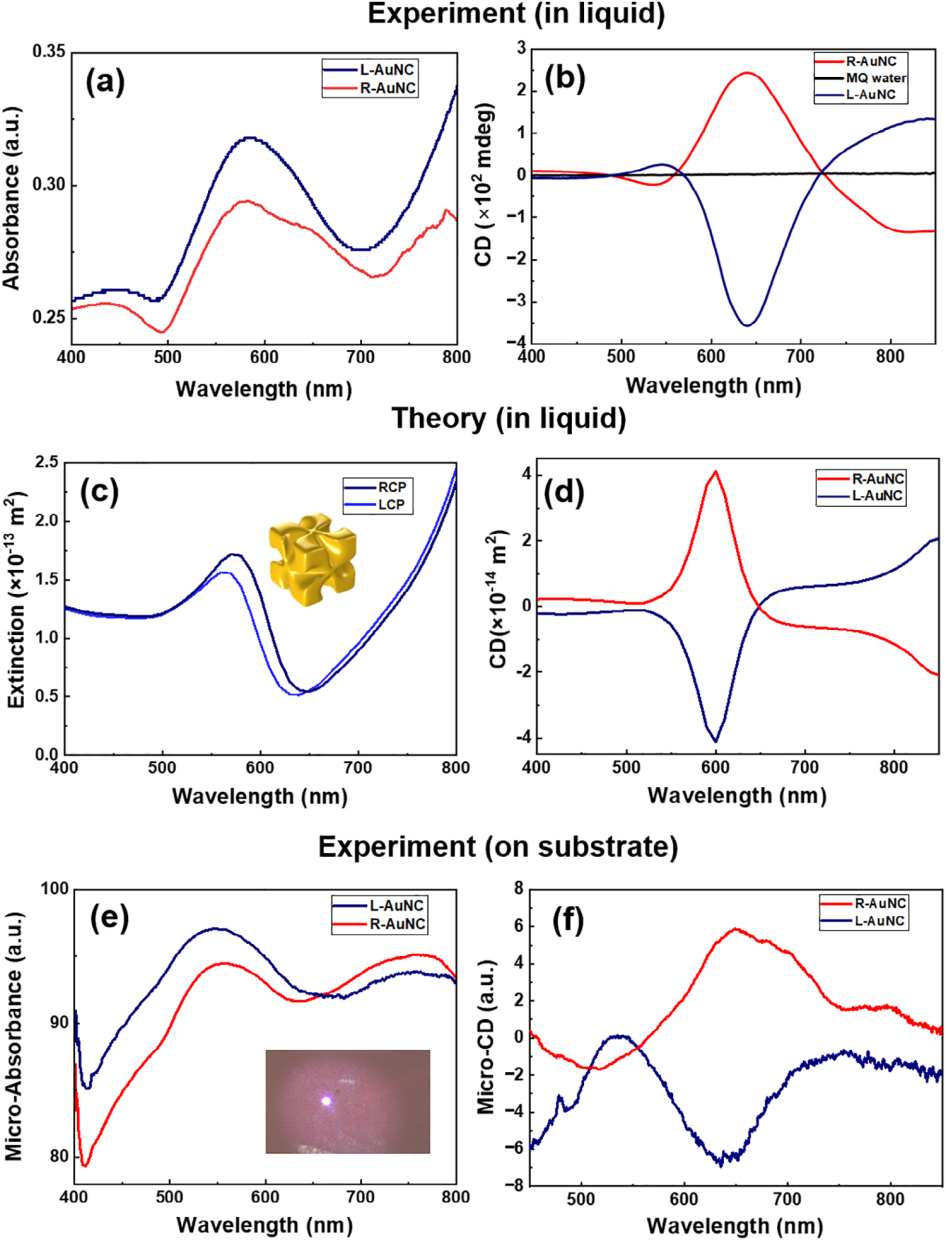
Optical properties of L‐ and R‐AuNCs a) UV–vis absorbance spectra b) optical CD spectra in liquid phase. c) simulated extinction and d) simulated CD in liquid phase. e) Micro‐absorbance on Si substrate. Inset is a representative optical image of the sample under 20× microscope objective. f) Micro‐CD of clusters deposited on Si substrate.

**Figure** **2**a,b present the absorbance and CD spectra of the chiral AuNCs in colloidal suspension. The particles exhibit a prominent localized surface plasmon resonance (LSPR) band centered ≈600 nm. Minor differences between L‐ and R‐AuNCs optical spectra are attributed to variations in the enantiopurity of the chiral ligands—specifically, L‐glutathione (GSH) and D‐glutathione used during synthesis. In particular, the lower purity of the D‐GSH employed in R‐AuNC synthesis may have introduced slight inhomogeneities in shape and yield, resulting in subtle differences in the optical responses.^[^ ^38^ ^]^ Additionally, the absorbance intensity is also influenced by the particle concentration during measurement. The optical CD and g‐factor are defined as:

(3) $$CD_{optical}=A_{LCP}-A_{RCP}$$

(4) $$g_{optical}=\frac{A_{LCP}-A_{RCP}}{(A_{LCP}+A_{RCP})/2}$$

where A_LCP_ and A_RCP_ are absorbance values under LCP and RCP light, respectively. At the CD maximum near 640 nm, the optical g‐factor was determined to be 0.18, consistent with previous reports.^[^ ^11^ ^]^ The experimental results are corroborated by electromagnetic simulations of extinction and CD spectra shown in Figure 2c,d. Simulated spectra under both liquid‐phase and substrate‐supported conditions (Figure S4, Supporting Information) reveal qualitative agreement with experimental measurements. It is important to note that the spectral shifts observed after drop‐casting the AuNCs onto silicon substrates are influenced not only by nanoparticle aggregation but also by the dielectric properties of the substrate itself. This is evident from a direct comparison of the chiroptical spectra in solution and on silicon (Figure S4, Supporting Information), which shows a clear redshift in resonance. COMSOL simulations further support this observation, demonstrating modified near‐field distributions and altered plasmonic resonances due to the substrate, as shown in Figure S5 (Supporting Information). Notably, both experiment and theory show the emergence of a second CD peak (or dip) in the near‐infrared (NIR) region—≈850 nm in solution and 720 nm on Si substrate—suggesting a reversal of the CD sign at longer wavelengths. This reverse CD feature reflects the complex multipolar plasmonic modes supported by the chiral AuNCs and highlights the influence of the substrate on the chiroptical response.

**Figure 3:**
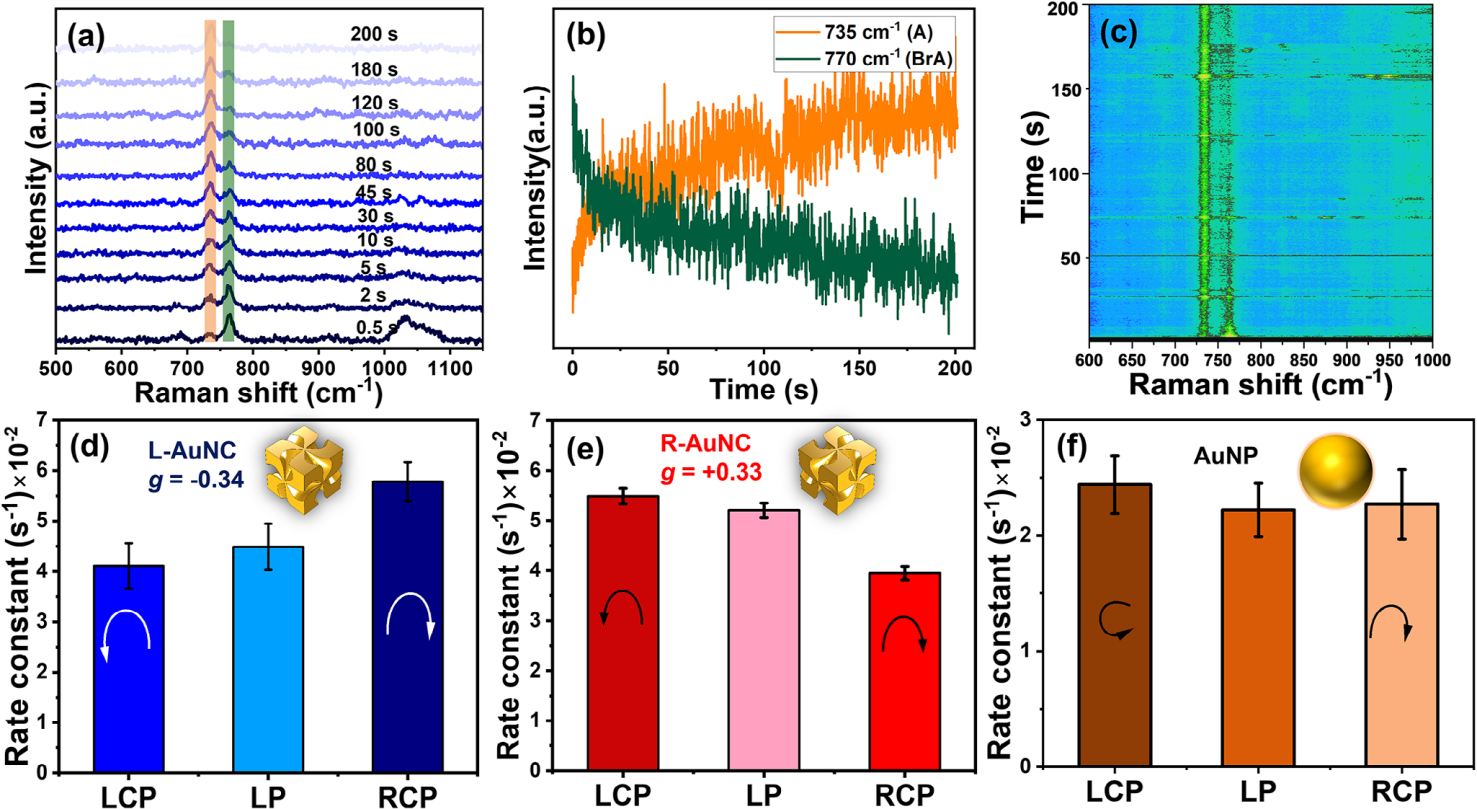
Plasmon‐induced transformation of BrA into Adenine a) SERS spectra of BrA at different points of time, the most significant change in spectra is highlighted b) Kinetics of ring breathing modes intensity of reactant BrA (770 cm^−1^) and product Adenine (A) (735 cm^−1^), c) the 3‐D contour plot of the temporal evolution of reaction under SERS d,e) reaction rate constants at different polarization states of incident light with L‐AuNCs and R‐AuNCs f) Control experiment on achiral Au 60 nm nanospheres with laser power 1.5 mW. Reaction rates were determined by the intensity decay curve of BrA ring breathing vibration (770 cm^−1^) at 633 nm excitation and laser power was 0.5 mW.

**Figure 4:**
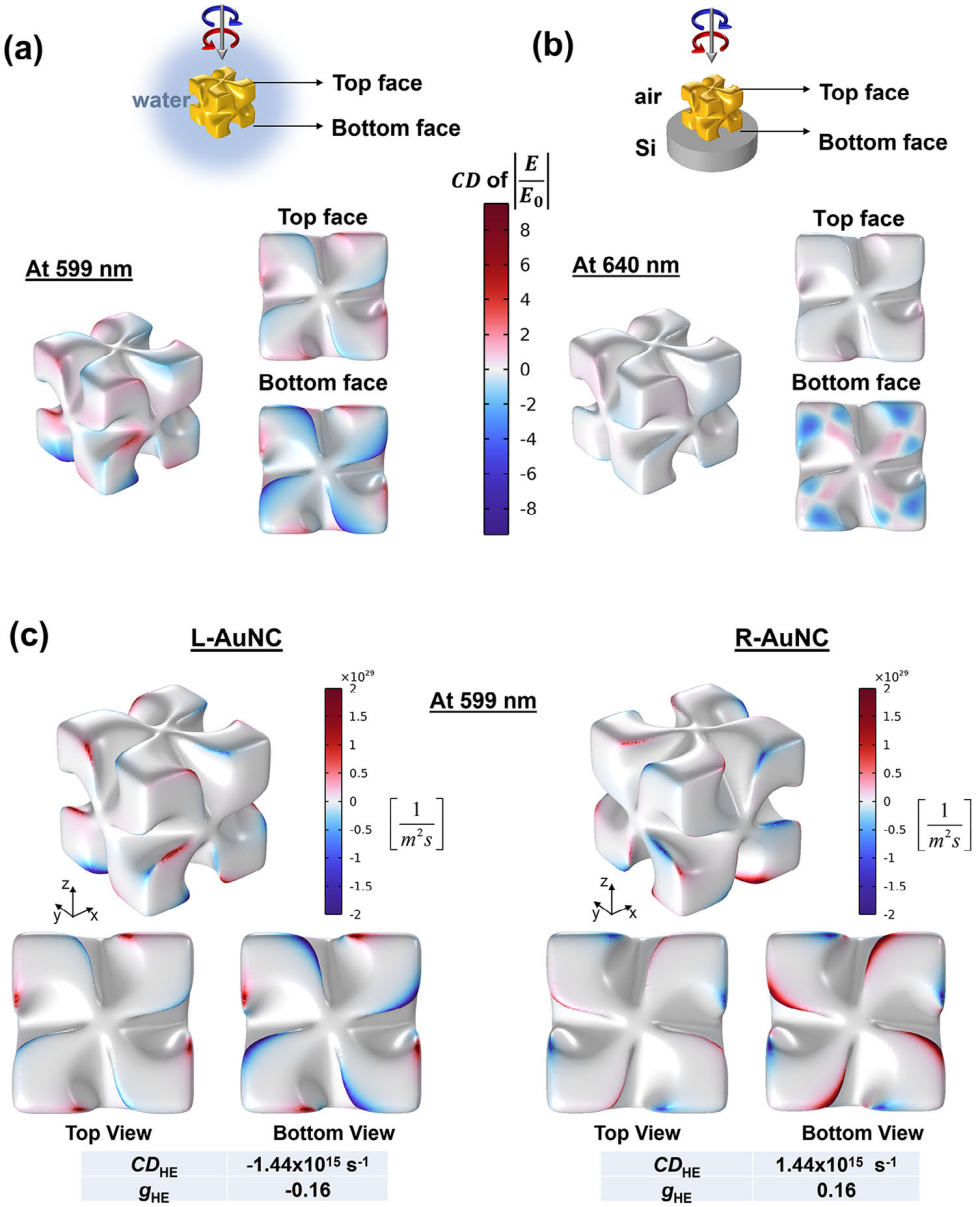
a) Chiral hot spots localization for L‐AuNCs in water solution and b) on a Si substrate surrounded by air. Each case was simulated for the most important CD resonance from panels (b,e) from Figure S4 (Supporting Information). c) Hot electron generation CD_HE_ maps of a single L‐AuNC and R‐AuNC in solution at 599 nm excitation calculated using COMSOL. The table below each figure shows the corresponding CD_HE_ values and *g*_HE_‐factors obtained from their respective rate of hot electron generation integrated over the entire surface of the structure. All simulations were carried out for an incoming light intensity of 3×10^7^ W cm^−2^ and the CPL illumination along the ‐k//z direction, such that the incoming light hits the top face of the helicoid.

The ≈600 nm CD band arises from the combination of electric dipole (ED), electric quadrupole (EQ), and magnetic dipole (MD), whereas the ≈850 nm peak is primarily attributed to the ED interactions.^[^ ^39^, ^40^ ^]^ To enable SERS‐based reaction tracking, nanoparticle clustering is essential to form intense hot spots where the local electric field is strongly confined and enhanced. However, both the absorbance and CD response are highly sensitive to the spatial arrangement of chiral structures on surfaces. For example, chiral AuNCs deposited on a Si substrate exhibit a blue shift in the absorbance spectrum (Figure 2a,e; Figure S4d, Supporting Information). A similar blue shift is observed in the CD band (Figure S6a, Supporting Information), attributed to the lower refractive index of air compared to aqueous environments.^[^ ^31^, ^41^ ^]^ Conversely, dispersion of AuNCs in higher refractive index media leads to redshifts in the CD spectra (Figure S6b, Supporting Information). To induce aggregation, AuNCs were randomly drop‐cast onto Si substrates, resulting in shifted absorbance maxima and the emergence of new spectral features in both absorbance and CD spectra (Figure 2e,f). Despite these variations, micro‐CD measurements of the aggregated AuNCs show a dominant CD peak at ≈640 nm, supporting the use of 633 nm excitation for probing photochemical chirality via SERS (**Figure** **3**). Figure S5 (Supporting Information) further explores the spatial distribution of hot spots under various resonance excitations, revealing asymmetric CD enhancement of the local electric field—typically localized on opposing nanoparticle faces under irradiation. For L‐AuNCs in solution, the hot spots are predominantly negative across all faces (assuming isotropic illumination), consistent with the ≈599 nm CD minimum in Figure 2d. On Si substrates, multiple multipolar resonances appear at the Au–Si interface (Figure S4e, Supporting Information), with the CD band shifting from 599  to 640 nm (Figure S4b,e, Supporting Information), aligning well with experimental trends shown in Figure 2. Excitation at 640 nm (close to the laser wavelength used) produces a negative CD in the field enhancement (**Figure** **4**a,b), which correlates with both the hot‐electron generation CD (Figure 4c) and the experimental photochemical CD (Figure 3).

The plasmon‐driven dissociative electron transfer from chiral AuNCs to achiral molecule BrA was monitored under dry conditions at room temperature in real‐time using SERS. This was achieved by tracking the ring breathing modes at 770 cm⁻¹ (BrA) and 735 cm⁻¹ (adenine, A), as shown in Figure 3a–c. The reaction progresses rapidly – the product A appears in the first scan, with near‐complete conversion observed within 200 s, indicated by the disappearance of the BrA peak. The time‐dependent SERS spectra (Figure 3a) and the corresponding contour map (Figure 3c) clearly depict the decay of BrA and the growth of A. Reference Raman and SERS spectra of BrA and A are provided in Figures S7 and S8 (Supporting Information). Control experiments (Figure S9, Supporting Information) confirm that the reaction does not proceed in the absence of plasmonic NPs. Due to the high reaction rate, the A peak is evident even in the first scan. While the rate can be modulated by adjusting laser power, excessively low power also weakens the SERS signal, complicating detection (Figure S10, Supporting Information). To better capture early‐stage kinetics, experiments were repeated using 60 nm spherical Au NPs, as the chiral AuNCs react too quickly to obtain a pure BrA spectrum at t = 0. Reaction kinetics were analyzed by applying fractal‐like kinetics to account for the heterogeneous distribution of reactive sites:

(5) $$\ln\left(\frac{I_{0}}{I}\right)=\frac{k}{1-h}t^{1-h}$$

*I*_0_ is the initial intensity of BrA ring breathing mode at *t* = 0, *I* is the intensity at time *t* and *h* is the fractal dimension.^[^ ^33^, ^34^, ^42^ ^]^

In this study, both LP and CP light were used to initiate plasmon‐driven reactions on chiral AuNCs. Under 633 nm laser excitation, L‐AuNCs exhibited a significantly higher reaction rate with RCP light compared to LCP and LP light, yielding a photochemical g‐factor (𝑔_PC_) of −0.34 (Figure 3d). The corresponding rate constants for all polarization conditions, as shown in Figure 3d,e, are summarized in Table S1 (Supporting Information).

Analogous to the g‐factor used in chiroptical spectroscopy (*g*_optical_), defined in Equation (4), the photochemical g‐factor (𝑔_PC_) quantitatively describes the differential reaction efficiency under LCP and RCP illumination. By replacing optical absorbance with reaction rates, the photochemical g‐factor is defined as

(6) $$g_{PC}=\frac{k_{LCP}-k_{RCP}}{\left(k_{LCP}+k_{RCP}\right)/2}$$

where *k*_LCP_ and *k*_RCP_ are reaction rate constants under LCP and RCP illumination.

The sign of the photochemical g‐factor (𝑔_PC_) correlates with the optical circular dichroism (CD) and optical g‐factor of the chiral AuNCs. Notably, the magnitude of 𝑔_PC_ is nearly twice that of the corresponding optical g‐factor, reflecting an enhanced sensitivity of the reaction kinetics to light polarization. A similar trend was observed for right‐handed AuNCs (R‐AuNCs), where LCP illumination resulted in a higher reaction rate than RCP, yielding a positive 𝑔_PC_ of +0.33 (Figure 3e). The absolute rate of reaction for R‐AuNCs was comparable to that of L‐AuNCs. For L‐AuNCs, the reaction rate under linearly polarized light (*k*_LP_) was approximately the average of the rates under LCP and RCP illumination, within experimental uncertainty. However, for R‐AuNCs, some deviation from this average was observed (see Table S1, Supporting Information).

All reported data represent averages of five independently measured rate constants from distinct but equivalent areas on the substrate, each exhibiting uniform and moderate particle coverage. Laser power at the sample was carefully measured for each polarization state. As quarter‐wave plates do not alter light intensity, the incident power under LCP, RCP, and LP conditions was identical.

As a control, the experiment was repeated using achiral 60 nm gold nanospheres (AuNPs). Due to their lack of chiroptical activity, these nanospheres exhibited polarization‐independent reaction rates, with a photochemical g‐factor of 0.04 ± 0.02 (Figure 3f). Additional measurements on spherical AuNPs are shown in Figure S11 (Supporting Information). In contrast, the chiral AuNCs exhibited more than double the reaction rate of the spherical nanoparticles. This enhancement is consistent with theoretical predictions of the hot‐electron generation rate (Figure S12, Supporting Information), and can be attributed to several structural factors: the anisotropic shape of the nanocubes, their sharp edges and helicoidal grooves, and their larger overall size – all of which contribute to the formation of intense hot spots.

Within aggregates, AuNCs interact through various configurations, such as face‐to‐face and face‐to‐edge contacts, generating a high density of electromagnetic hot spots. These not only enhance the Raman signal but also significantly boost the rate of hot electron generation and transfer.

To investigate the role of helical morphology in shaping the optical properties of chiral nanocubes and its contribution to chiroptical activity, we performed numerical simulations of the optical response for a series of nanocube geometries—ranging from ideal, achiral cubes to fully developed chiral helicoidal cubes. Simulations were conducted for two representative sizes: 150 nm and 190 nm. The results, presented in Figure S13 (Supporting Information), highlight the critical influence of edge morphology on the position of the CD signals; specifically, the introduction of rounded edges significantly alters the spectral features. Furthermore, a pronounced shift in both extinction and CD spectra is observed when transitioning from achiral to chiral structures, underscoring the emergence of optical activity due to morphological asymmetry. These findings suggest that achiral cubes do not serve as more appropriate reference systems than smaller spheres.

Hot‐electron (HE) generation maps for L‐AuNCs were calculated using COMSOL Multiphysics under LCP and RCP light excitation (see Methods section for details). Figure 4c displays the HE generation circular dichroism (CD_HE_) maps for individual L‐ and R‐AuNCs at the main simulated resonance wavelength of 599 nm (see Figure 2d). The calculated signs and magnitudes of the g‐factors (*g*_HE_) show excellent agreement with experimental photochemical g‐factor values, validating the predictive power of the simulations. Additional simulations of other resonant modes and the influence of the substrate are presented in Figures S14–S17 (Supporting Information). The HE maps closely follow the absorbance behavior and optical CD of the sample, confirming the underlying plasmonic origin of chiral asymmetry. Analogous to optical CD and g‐factors defined in Equations (3) and (4), CD_HE_ and g_HE_ provide quantitative measures of chiral HE generation efficiency.

**Figure 5:**
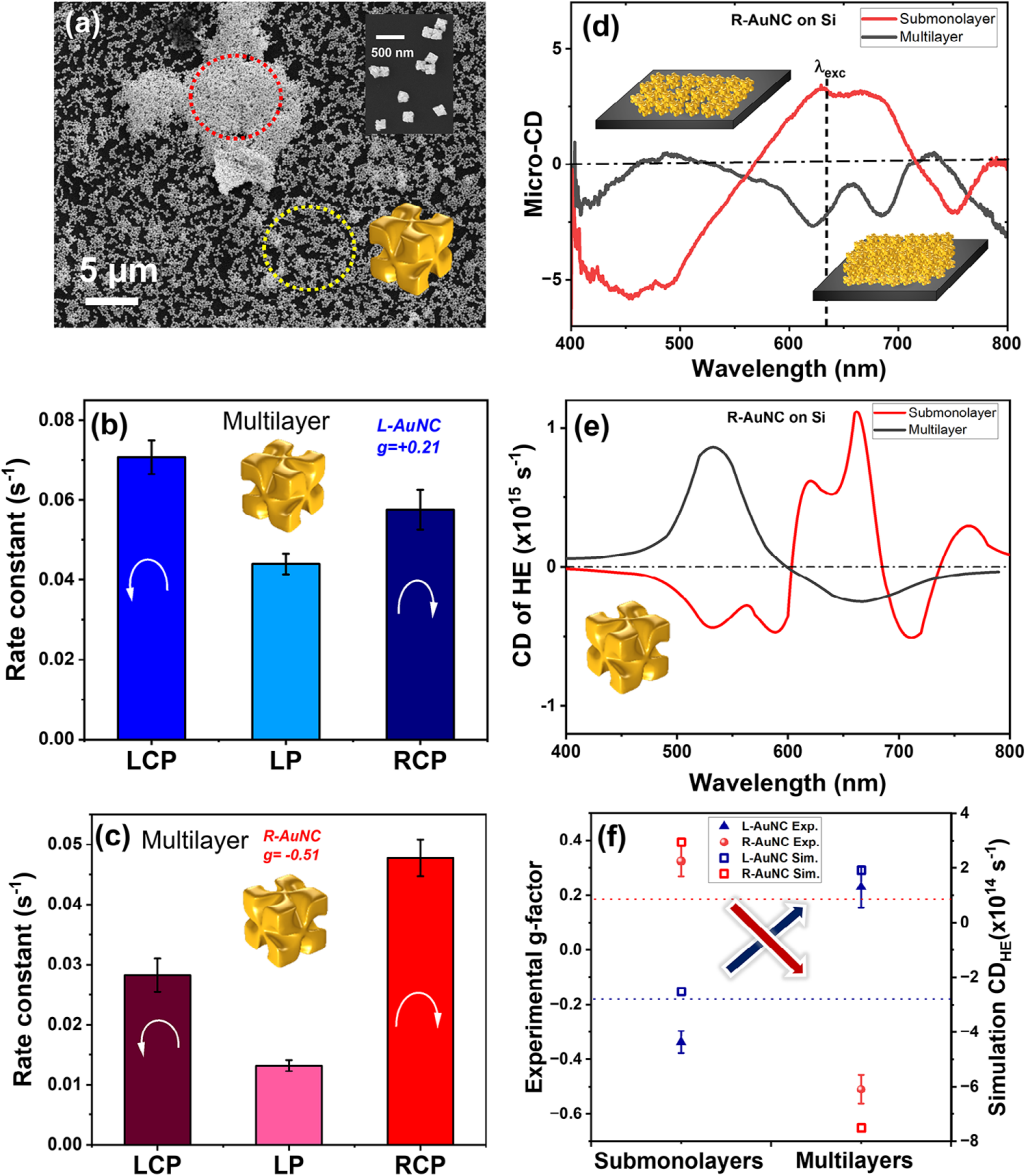
Reversal of CD and g‐factor on multilayers of AuNCs: a) SEM images of the substrate; highlighting multilayer (red circle) and submonolayer (yellow circle) coverage, rate constants under different states of light with b) L‐AuNC and c) R‐AuNC. In both cases linearly polarized light shows the lowest rate constant d) experimental micro‐CD (optical) spectra e) simulated CD_HE_ spectra on submonolayer and multilayer coverages of R‐AuNC f) comparison of photochemical *g*‐factors on submonolayers and multilayer coverage, the red and blue dotted horizontal lines indicate the value of optical g‐factors for R‐and L‐AuNCs, respectively. The measurements were carried out at 0.5 mW of 633 nm laser excitation. Simulated CD_HE_ for both cases of AuNC's densities on a silicon substrate are shown. From submonolayer to multilayer areas on the substrate, reversal of CD and g‐factor observed at excitation wavelength. See Figures S18 and S19 (Supporting Information) for full simulations details.

Importantly, the chiroptical response was found to be highly sensitive to the aggregation state of the AuNCs, yielding unexpected behaviors that can be leveraged for chiral amplification. Two distinct surface coverage regimes were investigated: i) submonolayers, and ii) multilayered assemblies of chiral AuNCs, as marked by the yellow and red circles in the SEM image in **Figure** **5**a. Complementary AFM measurements (Figure S1, Supporting Information) confirmed the stepwise increase in surface height corresponding to the number of stacked layers.

Regions with multilayered coverage exhibited significantly stronger Raman signals, higher signal‐to‐noise ratios, and markedly improved reaction kinetics. This enhancement is attributed to intense local electric fields generated at the densely packed hot spots formed in random aggregates of chiral AuNCs. Strikingly, a reversal in the sign of the photochemical g‐factor (*g*_PC_) was observed at the 633 nm excitation wavelength: for L‐AuNCs, the reaction rate *k*_LCP_ exceeded *k*_RCP_, yielding a positive *g*_PC_ of +0.21 (Figure 5b); for R‐AuNCs, the opposite behavior was observed in multilayered regions, with *k*_LCP_ < *k*_RCP_, resulting in a negative *g*_PC_ of −0.51 (Figure 5c). This inversion coincided with a reversal of the optical CD signal at 633 nm, as seen in experimental micro‐CD measurements (Figure 5d), and is further supported by simulated CD_HE_ spectra for different aggregation states (Figure 5e). The inversion of the chiral signal observed in multilayered assemblies is attributed to strong inter‐nanocube (NC) coupling and the pronounced sensitivity of CD to subtle changes in the plasmonic system's architecture. COMSOL simulations reveal that periodic stacking of NCs induces CD inversion as a result of near‐field interactions between adjacent particles. This interparticle coupling alters the local electromagnetic environment and modifies the resonance conditions of the chiral modes. Furthermore, an effective medium approximation provides complementary qualitative insight: increasing the real part of the surrounding dielectric function can also lead to a reversal of the CD signal. Together, these findings underscore the critical role of both nanoscale arrangement and dielectric environment in dictating the chiroptical response of plasmonic nanostructures.

A side‐by‐side comparison of experimental *g*_PC_ values and simulated CD_HE_ in submonolayer versus multilayer regions is shown in Figure 5f. Interestingly, reaction rates under LP light (*k*_LP_) were consistently lower than those under circular polarization, which we attribute to nonlinear coherent interference effects involving randomly mixed polarization states in the chiral medium.

Due to the inherent difficulty in resolving the exact spatial arrangement of individual nanoparticles (NPs) within experimental samples, we simulated a range of configurations—from isolated single particles to densely packed assemblies—to capture the influence of structural variation on chiroptical behavior (see Figure S18, Supporting Information for detailed schematic). Within each regime, multiple configurations were averaged, and the resulting CD_HE_ values for submonolayer and multilayer conditions are presented in Figure 5b, with comprehensive data in Figure S19 (Supporting Information). A clear inversion of the CD_HE_ sign is observed when transitioning from the single‐particle limit to densely packed structures. This shift is primarily driven by the anisotropic distribution of plasmonic hot spots and significant modifications in the local dielectric environment. Additional simulations—spanning absorbance, scattering, extinction, CD spectra, and HE maps for isolated particles and dimers across various excitation wavelengths—are shown in Figures S20–S24 (Supporting Information). Overall, these effects can be attributed to changes in the system's effective permittivity. To explore this further, we simulated the influence of the embedding medium's effective permittivity on the CD and g‐factor (Figure S25, Supporting Information). As shown in Figures S25a,b (Supporting Information), both CD absorbance and CD_HE_ spectra evolve with increasing permittivity. Notably, Figure S25c (Supporting Information) reveals a multi‐resonant behavior in the photochemical g‐factor, while Figure S25d (Supporting Information) illustrates a sign inversion—from negative to positive—when the effective permittivity increases from 1.8 to ≈3, under excitation at 600  and 633 nm, respectively.

**Figure 6:**
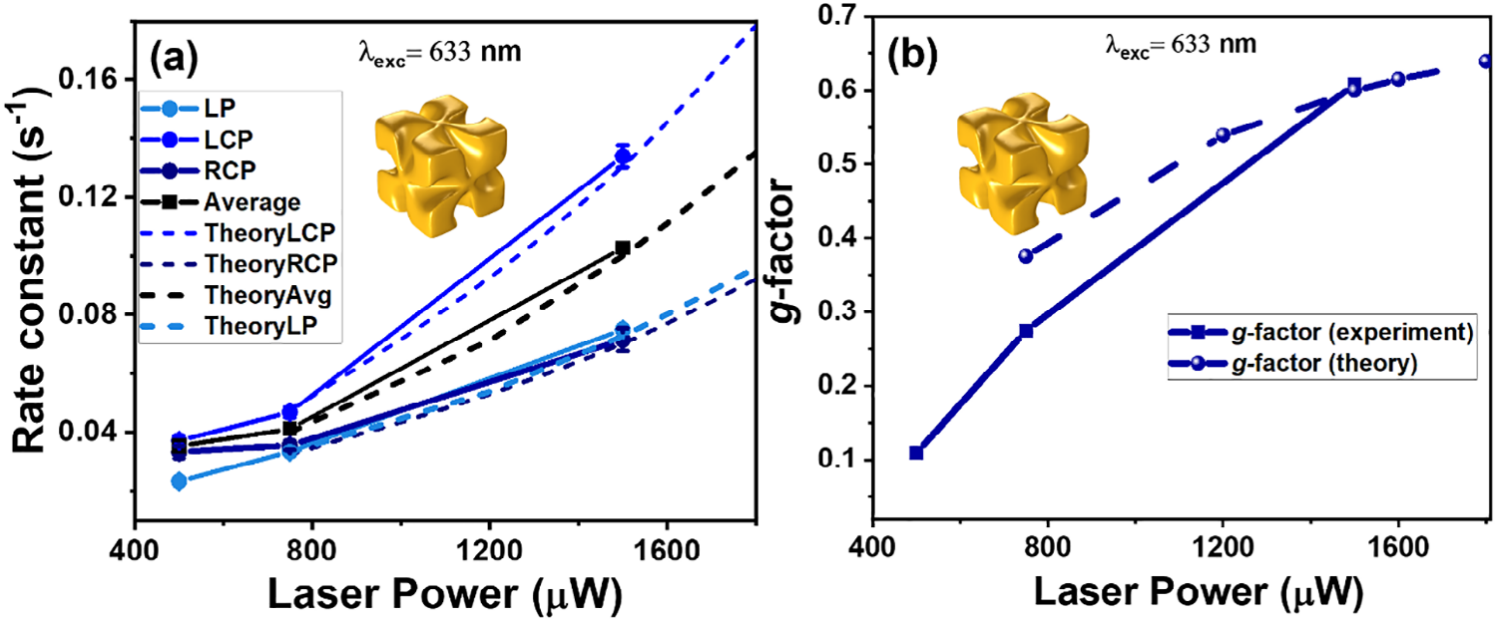
Laser power dependence of plasmon‐driven reaction kinetics on multilayered L‐AuNCs substrates at 633 nm excitation: a) Reaction rate constants as a function of polarization state and excitation power. b) Photochemical g‐factor versus laser power.

The power dependency of rate constants on thick layers of chiral L‐AuNCs was investigated at a laser wavelength of 633 nm, revealing a different behavior for the different polarizations (**Figure** **6**a), which provides the possibility to boost the g‐factor by means of excitation power (Figure 6b).

The power dependence of reaction rate in a plasmon‐induced reaction depends on the specific reaction under consideration and is determined by the mechanism of reaction. A purely hot‐electron‐induced reaction is supposed to show a linear power‐dependence of the reaction rate while a temperature‐dependent reaction is expected to show an exponential rise of the reaction rate with the power.^[^ ^23^, ^43^ ^]^ In previous work using spherical Au particles, it was shown that the debromination of BrA is predominantly hot‐electron‐driven,^[^ ^32^, ^34^ ^]^ but there is a deviation from the linear power‐dependence at higher powers due to stronger contributions of heat. With the chiral nanocubes the reaction rates are significantly higher than the ones obtained with spherical particles (Figure 3d–f), and the data suggest that the non‐linear regime is reached already at lower laser powers than observed previously for spherical particles. Figure 6a shows that CP excitation shows already deviations from a linear power‐dependence between 500 and 1500 µW, which is more pronounced for LCP than for RCP (in the case of L‐AuNCs). This in turn results in an increase of the g‐factor. As shown in Figure 6b, the g‐factor grows from ≈0.1 at 500 µW to ≈0.6 at 1500 µW, exceeding the optical g‐factor by a factor of ≈3. This power‐dependent amplification of chiral selectivity is a key result of our study, suggesting that nonlinear plasmonic effects in chiral assemblies can be harnessed to boost enantioselective photochemistry.

The power‐dependence of rate constants can be described with a simple phenomenological model of the system, which is both chiral and isotropic. The most general form of the expansion for the rate constant at small and intermediate powers is:

(7) $$\begin{array}{ccc} k\left(P_{LCP},P_{RCP}\right) & = & \alpha_{-}P_{LCP}+\alpha_{+}P_{RCP}\\ & + & \beta \left(P_{LCP}^{2}+2\gamma_{1}P_{LCP}P_{RCP}+\gamma_{2}P_{RCP}^{2}\right) \end{array}$$

where *P*_*LCP*(*RCP*)_ are the fluxes. Here, the coefficients in Equation (7) are empirical constants, which should be found from the fitting to the experiment. γ_1_ and γ_2_ are dimensionless nonlinear expansion parameters for chiral media and media in cylindrical 2D rotational symmetry, which should be computed from a microscopic theory. Figure 6a includes the modeling results computed in this manner at high power, keeping only the nonlinear terms in Equation (7). Interestingly, just with two fitting parameters (γ_1_ =  0.25 and  γ_2_ =  0.45), we are able to describe the sequence of the rates for multilayers of L‐AuNCs (i.e., that $k_{LCP}>k_{RCP}∼k_{LP}$) and show that the case of $k_{LP}<\;{\bar{k}}_{CP}=\;\left(k_{LCP}+k_{RCP}\right)/2$ is physically feasible in the nonlinear regime, whereas the linear regime should always have $k_{LP}={\bar{k}}_{CP}$. Moreover, our model in the non‐linear regime predicts qualitatively the increase of the g‐factor at high powers (Figure 6b).

## 3 Conclusion

We investigated asymmetric hot‐electron induced photochemistry on chiral plasmonic substrates under polarized radiation. Chiral AuNCs (432 helicoids III) with an average side length of 180 nm exhibit high optical activity (g‐factor = 0.18) and significant electric field enhancement through LSPR. Consequently, SERS was employed to trigger and monitor a chemical reaction on the surface of these cubes in real‐time.

The model reaction for hot‐electron‐assisted plasmonic chemistry involved the dehalogenation of a halogenated nucleobase, BrA. The cleavage of the C‐Br bond was efficiently monitored through time‐dependent SERS under different polarized states of incident continuous‐wave laser light. The reaction rate was determined by analyzing the decay curve of the Raman intensity of the reactant. Control measurements were conducted using achiral spherical gold nanoparticles. The reaction was examined in a dry state on aggregates to achieve a pronounced SERS effect, thereby enhancing the Raman signal of the reactant molecule over the exposure time.

We utilized excitation wavelengths of 633  and 785 nm, observing a pronounced effect of CPL on the rate constants at 633 nm. The reaction was effectively observed with the 785 nm laser as well (see Figures S26 and S27, Supporting Information), though the rate constants were five times lower than those with the 633 nm laser (Figure S28, Supporting Information) and no significant preference for a specific polarization was observed. In multilayer cases, the power dependency at 785 nm showed a significantly lower g‐factor compared to 633 nm excitation, with noticeable fluctuations. At submonolayer coverage of AuNCs the dependence of rate constants on the polarization of incident light at 633 nm is consistent with the optical activity (CD_optical_) of the chiral structures. Interestingly, we found that the photochemical g‐factor (defined by the rate constants with LCP and RCP excitation) is highly dependent on the coverage of AuNCs on the substrate. Accordingly, on multilayers of AuNCs a reversal of photochemical g‐factor was observed with a non‐linear power‐dependence of rate constants, which leads to the possibility of boosting the photochemical g‐factor to values as high as three times the optical g‐factor. Theoretical simulations performed using COMSOL, including extinction and hot‐electron generation maps, closely align with our experimental results and provide insight into the underlying mechanisms.

These findings open promising avenues for exploiting chiral plasmonic nanostructures in enantioselective photochemistry, catalysis, and sensing applications, where dynamic control over chiral reactivity can be harnessed.

## 4 Experimental Section

### Chemicals

BrA and A were purchased from Alfa Aeser. Ethanol and MQ water were used for solvent or washing reagents.

### Synthesis of chiral AuNCs (432 helicoids)

The synthesis of chiral AuNCs (432 helicoid III nanoparticles) involves a peptide‐directed growth process starting with 40 nm octahedral gold nanoparticles seeds.^[^ ^44^ ^]^ The growth solution was prepared by sequentially adding cetyltrimethylammonium bromide (CTAB) at a concentration of 100 mm (0.8 mL), hydrogen tetrachloroaurate (HAuCl_4_) at 10 mm (0.2 mL), ascorbic acid (AA) at 100 mm (0.475 mL), and L‐glutathione (L‐GSH) at 5 mm (5 µL) to deionized water. D‐GSH was used to synthesize R‐AuNCs. The growth reaction is initiated by injecting 50 µL of the octahedral seed nanoparticles into this solution. The mixture is then left undisturbed in a 30 °C bath for 2 h, during which the solution changes from transparent purple to blue with significant scattering. After the reaction, the growth solution is centrifuged at 1677×g for 60 s and washed twice with 1 mm CTAB to remove any unreacted reagents and residuals. Finally, the synthesized helicoids are redispersed in 1 mm CTAB. For the complete synthesis protocol of the AuNCs, readers are referred to the reference.^[^ ^38^ ^]^

### AFM

AFM measurements were conducted on a Bruker Multimode‐8 atomic force microscope from Billerica, Massachusetts, USA. Chiral nanocubes were dried onto a silicon substrate for examination. The Tapping mode was employed for imaging, utilizing a tip TAP150 with a resonant frequency of 150 kHz, enabling high‐resolution characterization of the sample surface.

### TEM and SEM

JEM 1011 from JEOL, Japan was used for acquiring transmission electron micrographs of chiral and non‐chiral nanoparticles. The operating voltage was 80 kV. For scanning electron micrographs (SEM), Zeiss, Ultra plus microscope was employed at 15 kV accelerating voltage.

### Absorbance and CD Measurements

For acquiring absorbance spectra in solution, Perkin Elmer spectrometer was utilized at 1 nm step‐size. Measurement of circular dichroism (CD) from chiral AuNC (432 helicoid III) was conducted on CD spectrometer (J‐815, Jasco) with a quartz cuvette of path length 1 mm.

### DLS and Zeta Potential

Malvern Instruments Ltd. Zetasizer was used for the DLS and Zeta Potential measurements.

### Substrate Preparation for SERS Measurements

The sample preparation involved initially subjecting colloidal AuNCs to centrifugation at 6000 RPM, followed by a thorough washing with high‐purity water (MQ) to eliminate any residual substances and surfactants. Subsequently, 1 mm solution of 8BrAdenine in ethanol‐water mixture (1:1) was mixed with the AuNC solution such that the final concentration of BrA in solution was maintained as 0.1 mm. Then the mixture was incubated at 350 RPM for the duration of 3 h at 30 °C to ensure full and uniform adsorption of the molecules onto the surface of the nanocubes. A final step included a single wash using an ethanol‐water mixture in a 1:1 ratio to eliminate any excess or unbound molecules. The concentrated solution was dropcasted onto the cleaned Si substrate. The coated chips were allowed to dry overnight at ambient conditions before measuring in Raman. Notably, the chiral morphology remained highly stable even after treatment and laser exposure (Figure S29a,b, Supporting Information). Zeta potential measurements (Figure S29c, Supporting Information) clearly indicated a reduction in surface charge after washing the particles with MQ water, as well as the subsequent substitution of residual CTAB with reactant BrA.

### Micro‐Absorbance and Micro‐CD

The micro‐absorption measurements were performed on a home‐built setup. The core of the setup is an inverse microscope from Olympus (IX71). As an excitation source, a supercontinuum laser (FIU‐15) from NKT Photonics was used. To generate circularly polarized light a polarizer and quarter waveplate from Thorlabs were placed in the beam path. The circularly polarized light was focused with a 20x objective from Olympus (NA 0.25) on the sample. The same objective collected the light reflected by the sample and guided it through a beam splitter to a fiber that was connected to an Avantes spectrometer. The micro‐absorbance spectra were measured at several spots on the sample. The reflectance of the Si substrate near the measurement area was used as I_reference_ for reflectance. $R=\frac{I_{sample}-I_{dark}}{I_{reference}-I_{dark}}\;$ was used to calculate the reflectance (R). The absorption (A) was calculated by A(%) = 100% – R(%) since the transmission on the silicon substrate was zero. For optical CD calculation, the formula in Equation (3) was used.

### SERS Measurements

The BrA coated AuNC samples were analyzed using a Witec alpha 300 Raman microscope equipped with 4 excitation lasers and polarization optics. Time‐series SERS measurements were performed using lasers at wavelengths of 633  and 785 nm, with varying power ranging from 200 µW to 2 mW with 50× objective (Olympus, Plan N, NA 0.75). The linear polarization was maintained through the optical fibers. A quarter wave plate (λ/4) was positioned before the microscope objective to generate CPL, including LCP and RCP light. The back scattered beam was collected by passing through an analyzer. This experimental setup allows the analysis of chiral nanocube clusters on the Si surface using Raman spectroscopy, with a specific focus on time‐series SERS spectra with CPL light. All SERS spectra were further processed using WITec Project 5.1 and Origin 9.1 software. Non‐linear allometric curve fitting was used for fractal kinetics data analysis.

### Determination of Rate Constants

Previous studies have shown that chemical hotspots correlate with optical hotspots, with charge carriers generated and transferred in these nanogaps. However, in colloidal aggregates, uneven hotspot distribution complicates quantitative SERS analysis, as most signals come from highly reactive hotspots, with minimal contribution from molecules outside these areas. Several studies have addressed this by considering plasmonic substrate inhomogeneity to analyze SERS‐monitored kinetics of plasmon‐driven photoreactions. The “fractal‐like kinetics”^[^ ^45^ ^]^ approach, accounting for reactive site inhomogeneity, fits well and can be applied to systems with limited reactant mobility, resulting in time‐dependent reaction rates. The underlying reaction showed fast rates, with most analyte molecules decomposing with in a few seconds under typical SERS settings.

The Raman intensity is directly proportional to the analyte concentration and concentration was replaced with the intensity. The reaction rate depends on the concentration of reactant [BrA] and hot electrons [hot e^−^] in the irradiated volume.

(8) $$Rate=-k\left[BrA\right]\left[hote^{-}\right]$$

Under continuous laser illumination, a constant equilibrium concentration of charge carriers was assumed because they are generated frequently, and their excitation and relaxation timescales are much shorter than those of the DEA reaction. In addition, the reaction coefficient here is time‐dependent, therefore wherever the term “Rate constant” was used, it refers to the “Rate coefficient” or *k*.

(9) $$k=k1t^{-h};0\leq h\geq 1;t\geq 1$$

Therefore, to accurately model the reaction kinetics, a 1st order fractal fitting was employed using the following equation:

(10) $$\ln\left(\frac{I_{0}}{I}\right)=\frac{k}{1-h}t^{1-h}$$

The curve fitting used here is:

(11) $$y=ax^{b}$$

(12) $$\ln\left(\frac{I_{0}}{I}\right)=at^{b}$$

Thus, the effective rate coefficient *k*  =  *a* · *b*

I_0_ is the intensity of BrA ring breathing mode at 770 cm^−1^ at *t* = 0, and I is denoted as intensity at time *t*. For the reaction kinetics, nonlinear allometric curve fitting was used in Origin9.1.An example fit for spherical gold nanoparticles (AuNPs) with a diameter of 60 nm is shown in Figure S11d (Supporting Information), where the fitting yielded a coefficient of determination R^2^ = 0.98, indicating excellent agreement with the first‐order fractal‐like kinetics model. All reported rate constants are averages over at least five measurements performed on independent but identical sample regions, with uncertainties given as standard deviations.

Due to random aggregation during drying on the substrate, the chiral nanoparticles were oriented randomly, potentially leading to fluctuations in reaction rates. This heterogeneity in aggregates is attributed to differences in local nanocrystal density and the distribution of hotspots between particles. As mentioned earlier, the drop‐casting method tends was employed to produce random particle aggregates, and these results confirm that the chiral optical and photochemical responses are highly sensitive to interparticle arrangement. While such stochastic assembly can hinder systematic mechanistic analysis, it offers a practical and straightforward route for substrate functionalization—particularly for exploiting plasmonic hot spots that are essential for strong SERS signals and efficient plasmon‐induced chemical reactions. Due to the cubic shape of the nanoparticles, some degree of order is established especially in the multilayers of cubes. extensive measurements across multiple samples and particle clusters were conducted, performing statistical analyses to distinguish consistent trends and mitigate variability. These experiments demonstrated high reproducibility in the photochemical circular dichroism, underscoring the robustness of these conclusions despite structural heterogeneity. During SERS measurements, care was taken to avoid re‐illuminating the same region of the sample. After each acquisition, the sample stage was slightly shifted to probe a fresh area while maintaining proximity to the previous spot. This approach ensured reproducibility while preventing photothermal or photochemical effects from prior laser exposure. Measurements were conducted on uniform, visually similar areas to facilitate the comparison of rate coefficients under different polarizations. Variations may also arise from irregularly shaped cubes, contributing to fluctuations in rate constants. A single SERS spectrum of BrA mixed with L‐AuNC was recorded using a 633 nm laser under both LCP and RCP illumination. The comparable intensities observed for both polarization states suggest that the reaction is primarily governed by charge transfer rather than near‐field effects (Figure S30, Supporting Information).

### Comsol Multiphysics Simulations

To model the optical properties and the hot‐electrons generation of the helical AuNCs, a commercial finite‐elements method (FEM) software (COMSOL Multiphysics software, with the RF module) was used. Several cases within mostly three different scenarios were stimulated: i) AuNCs in solution; ii) a single AuNC on a silicon substrate; and iii) several combinations of multiple AuNCs on a silicon substrate. All individual simulated AuNCs are of 190 nm side length, in order to follow experimental characterization sizes, and the Au's permittivity was taken from Johnson and Christy's.^[^ ^46^ ^]^ For the silicon substrate, Green's permittivity was used,^[^ ^47^ ^]^ for air ε_*air*_ =  1, for water ε_*water*_ =  1.8, and for the molecular coating ε_*coating*_ =  2.

Single systems, both in solution and on a substrate, were modeled with a typical absorption/scattering far‐field setup where a plane CPL‐polarized electromagnetic wave is incident on the NC, and a perfectly matched layer (PML) domain is outside of the media domain and acts as an absorber of the scattered field. Periodic systems were built as a periodic array of scatterers on a substrate, where a plane CPL‐polarized electromagnetic wave is incident on the array, with orthogonal incidence. The model first computes a background field from the plane wave incident on the substrate, and then uses that to arrive at the total field with the scatterer present.

### Optical Formalism

The NPs were illuminated with CPL light and calculated the optical response of the system. The formalism developed in this previous work was followed,^[^ ^48^ ^]^ where the incident electromagnetic field is defined as: ${\vec{E}}_{ext}=\;Re\left[{\vec{E}}_{0}e^{i\omega t}\right]$. By solving the Maxwell's equations within a classical framework, the calculations provide the far‐field quantities such the absorption (σ_*abs*_), scattering (σ_*scat*_), and extinction (σ_*ext*_) optical cross‐sections (σ_*ext*_ = σ_*abs*_  + σ_*scat*_). The scattering cross‐section is calculated by integrating the scattered intensity over a fictitious sphere around the NP, whereas the formalism for the absorption cross‐section is based on the following equations:

(13) Qabs=−ImεNPε0ω2∫dVE⃗ω·E⃗ω∗

(14) $$\sigma_{abs}=\frac{Q_{abs}}{I_{0}}$$

where *Q_abs_* is the absorbed power by the system, ε_*NP*_ is the dielectric constant of the metal nanoparticle, ω is the angular frequency of the incident light, ${\vec{E}}_{\omega }$ is the complex electric field inside the metal, and *I*_0_ is the photon flux magnitude (intensity for simplicity), given by

(15)

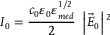

where ε_*med*_ is the dielectric constant of the medium, *c*_0_ is the speed of light in vacuum, ε_0_ is the vacuum permittivity, and $\left|{\vec{E}}_{0}\right|$ is the electric field magnitude of the incident electromagnetic wave.

### Hot‐Electron (HE) Generation Formalism

A HE generation formalism that is suitable for NPs in solution and on substrates was recently developed (see ref.[49, 50, 51]). In general, it has been shown that HE generation happens at the NC surface, driving local growth, etching, and other chemical transformations. Either the far‐field spectrum or local surface maps of the HE generation could be stimulated, the latter of great importance when NCs are anisotropic, as the case here with the helicoidal AuNCs. The HE spectrum reflects the absorption rate revealing the plasmonic resonances of the NC. The rates are computed for high‐energy electrons (E>Δ*E*_bar_), which can efficiently induce reactions at the surface or to be injected into a semiconductor scavenger, and are given by

(16) $$Rate_{\mathrm{HE},\mathrm{tot}}=\frac{1}{4}\times \frac{2}{\pi^{2}}\times \frac{e^{2}{E_{F}}^{2}}{\hbar }\frac{\left(\hbar \omega -\Delta E_{\mathrm{bar}}\right)}{{\left(\hbar \omega \right)}^{4}}\int_{S_{\mathrm{NC}}}{\left|E_{\omega ,\mathrm{normal}}\left(\theta ,\varphi \right)\right|}^{2}ds$$

where $E_{\omega ,\mathrm{normal}}\left(\theta ,\varphi \right)$ is the normal component of the electric field on the surface inside the NC; *E*_F_ = 5eV is the Fermi energy for gold. The integral is taken over the NP's surface; Δ*E*_bar_ = 1eV is the typical Au's injection threshold energy. Correspondingly, the local HE generation maps are calculated as

(17) $$Rate_{\mathrm{HE}}\left(r\right)=\frac{1}{4}\times \frac{2}{\pi^{2}}\times \frac{e^{2}{E_{F}}^{2}}{\hbar }\frac{\left(\hbar \omega -\Delta E_{\mathrm{bar}}\right)}{{\left(\hbar \omega \right)}^{4}}{\left|E_{\omega ,\mathrm{normal}}\left(\theta ,\varphi \right)\right|}^{2}$$

The CD and g‐factor for the HE generation with CPL are calculated in a similar fashion as in Equations (3) and (4), meaning:

(18) $$CD_{\mathrm{HE}}=\mathrm{Rat}e_{\mathrm{HE}}^{\mathrm{LCP}}\;-\mathrm{Rat}e_{\mathrm{HE}}^{\mathrm{RCP}}$$

(19) $$g_{HE}=\frac{Rate_{HE}^{LCP}-Rate_{HE}^{RCP}}{\left(Rate_{HE}^{LCP}+Rate_{HE}^{RCP}\right)/2}$$

## Conflict of Interest

The authors declare no conflict of interest.

## Supporting information



Supporting Information

## Data Availability

The data that support the findings of this study are openly available in Zenodo at https://doi.org/10.5281/zenodo.15550588.
