# Supplementary material for: Amplification of Photochemical Chiroptical Activity of Chiral Gold Nanocubes
Source: Small. 2025 Jun 22;21(36):2505093. doi: 10.1002/smll.202505093 (PMC12423909; doi:10.1002/smll.202505093)
Supplement: Supplementary file 1 — Supporting Information [file SMLL-21-2505093-s001.docx]

**Supporting Information**

**Amplification of Photochemical Chiroptical Activity of Chiral Gold Nanocubes**

*Shashank K. Gahlaut^a#^,* *Oscar Avalos-Ovando^b,c#^, Ryeong Myeong Kim^d^,* *Ridwan Hussein^b^, Sabrina Juergensen^e^, Stephanie Reich^e^, Alexander O. Govorov^b^, Ki Tae Nam^d^, Ilko Bald^a,f^**

^a^Institute of Chemistry, University of Potsdam, 14476 Potsdam, Germany

^b^Department of Physics and Astronomy, Nanoscale and Quantum Phenomena Institute, Ohio University, Athens, Ohio 45701, United States

^c^Department of Nanoscience, Joint School of Nanoscience & Nanoengineering, University of North Carolina at Greensboro, Greensboro, North Carolina 27401, United States

^d^Department of Materials Science and Engineering, Seoul National University, Seoul 08826, Republic of Korea

^e^Department of Physics, Freie University Berlin, 14195 Berlin, Germany

^f^Dynamics of Molecules and Clusters Department, J. Heyrovský Institute of Physical Chemistry of the CAS, Dolejškova 3, Prague, 18223, Czech Republic

*correspondence: [ilko.bald@uni-potsdam.de](mailto:ilko.bald@uni-potsdam.de)

^#^ Both authors contributed equally.

| 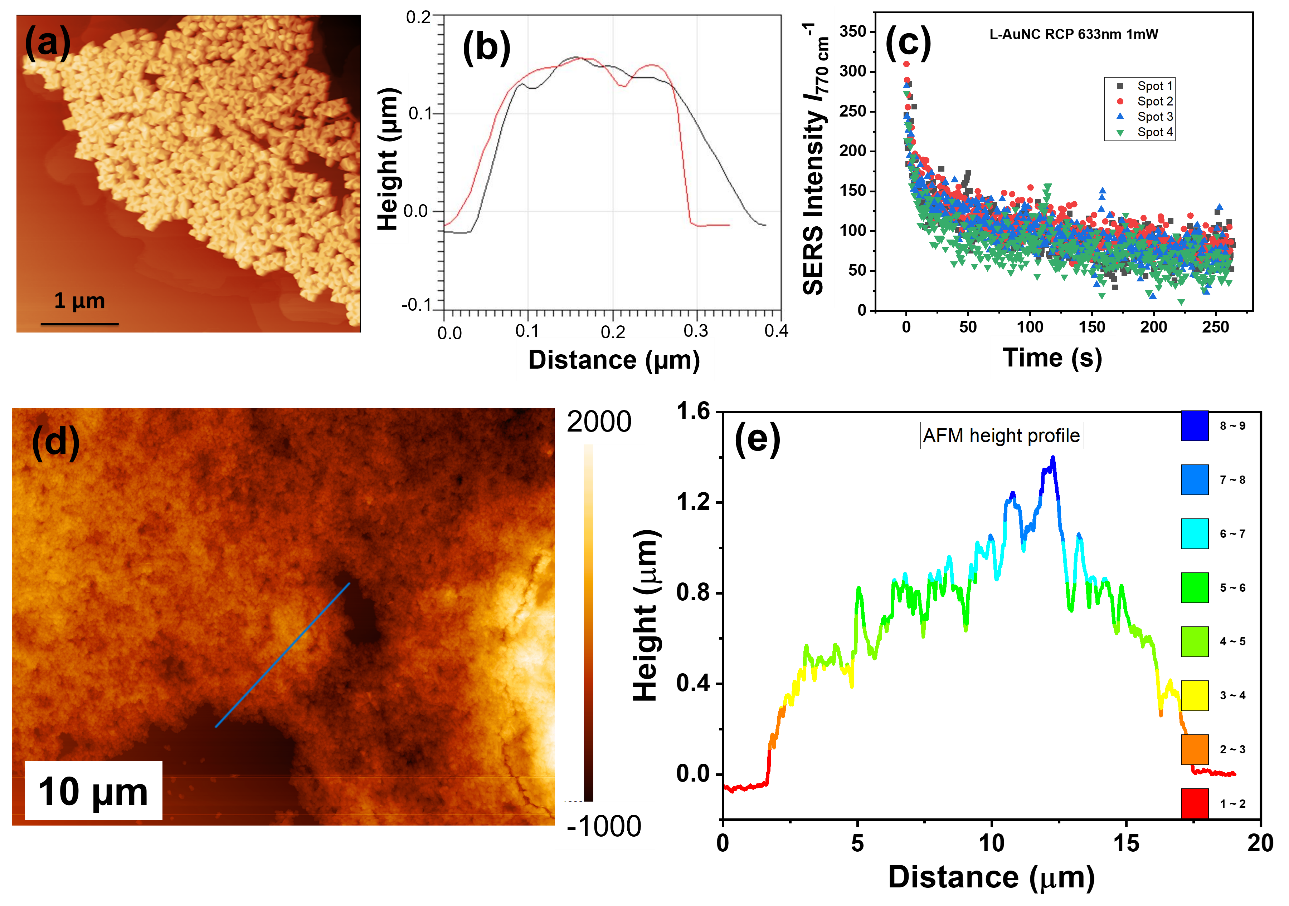 |
| --- |
| **Figure S1.** (a) AFM image of L-AuNCs (coated with reactant BrA) cluster dried on Si (b) Height profile of randomly chosen two single L-AuNCs and (c) SERS intensity profile of 770 cm^-1^ (decay of ring breathing mode of BrA) at four different spots on a cluster; represents the data reproducibility (d) Large area AFM scan (e) Height profile of an area under the blue line drawn in image (d); showing the different number of layers on the substrate. |

| 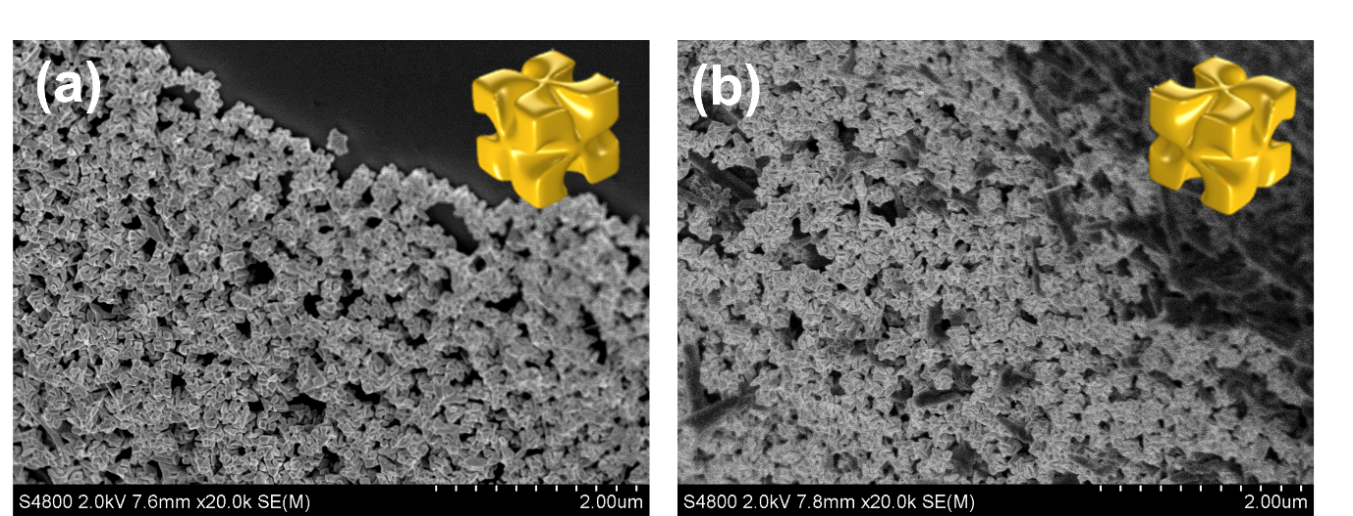 |
| --- |
| **Figure S2.** Scanning electron micrographs (SEM) of (a) L- (b) R- AuNCs aggregated on Si substrates. |

| 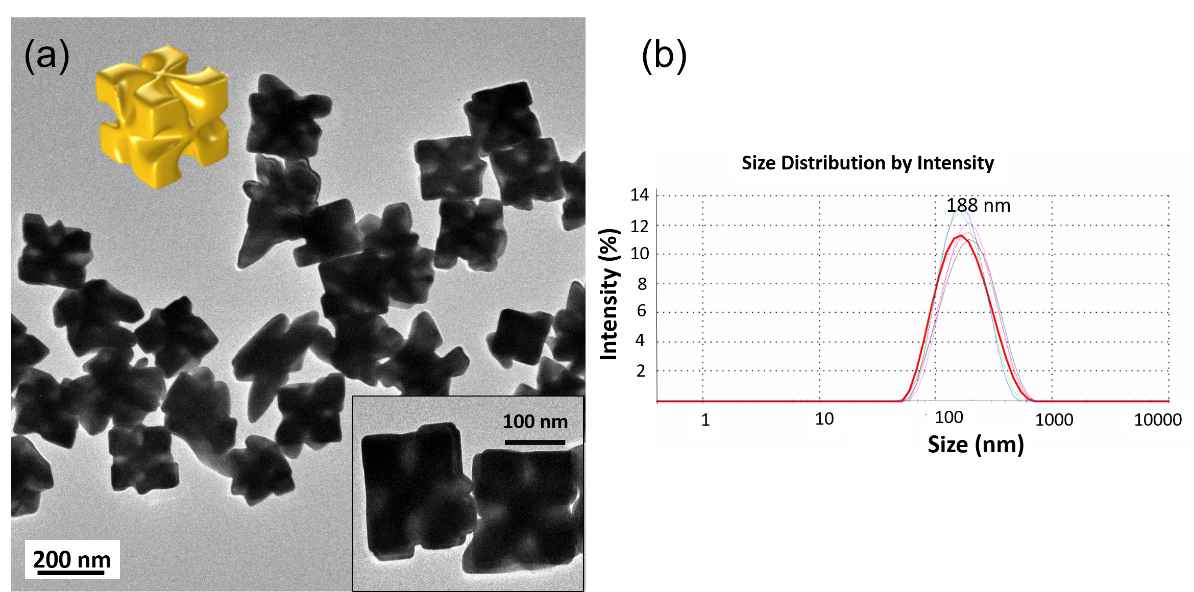 |
| --- |
| **Figure S3.** (a)Transmission electron micrographs (TEM) of L-AuNCs (b) Dynamic light scattering (DLS) spectra showing the size distribution of chiral cubes suspension in CTAB. |

| 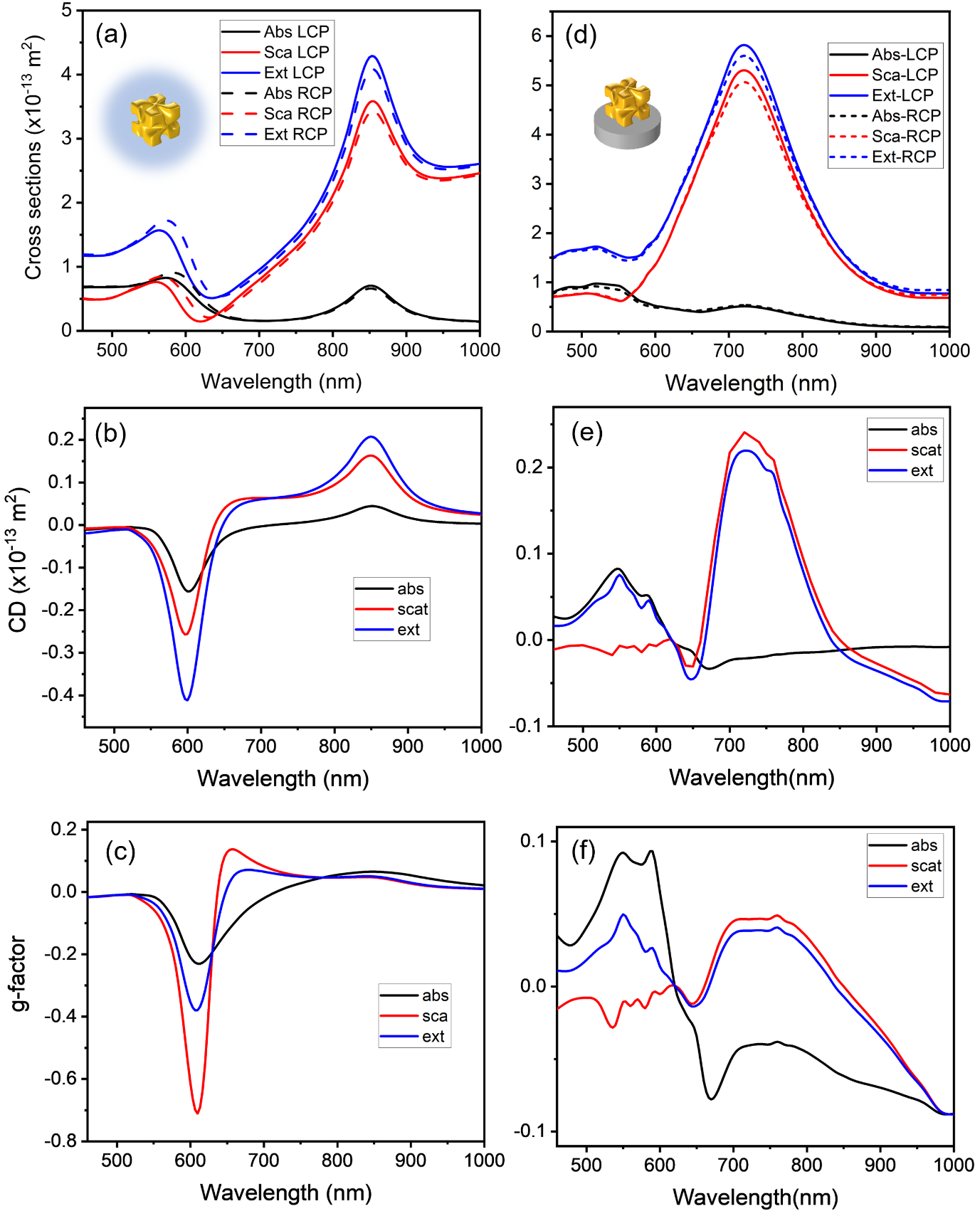 |
| --- |
| **Figure S4**. Simulated optical responses of the L-AuNCs chiral cubes in (a-c) water solution, and (d-f) on a Si-substrate with air environment. Panels (a,d) are the optical cross sections under CPL illumination; (b,e) are the CD spectra; and (c,f) are the g-factors. All simulations were carried out for an incoming light intensity of 3×10^7^ W/cm^2^ and the CPL illumination along the -k//z direction, such that the incoming light hits the top face of helicoid. |

| 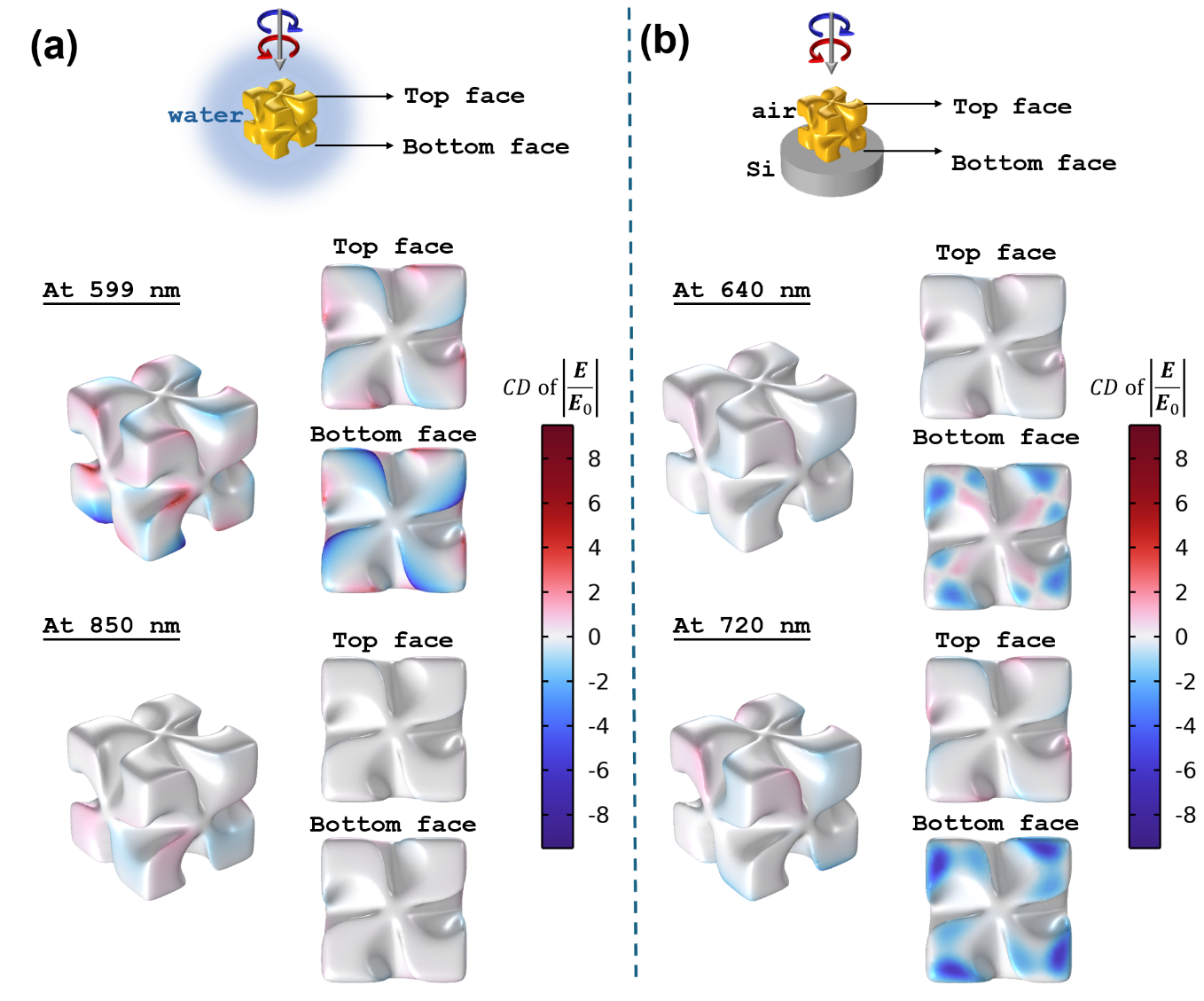 |
| --- |
| **Figure S5**. Simulated chiral hot spots localization for L-AuNCs (a) in water solution, and (b) on a Si substrate surrounded by air. Each case was simulated for the two most important CD resonances from panels (b) and (e) of Figure S4. All simulations were carried out for an incoming light intensity of 3×10^7^ W/cm^2^ and the CPL illumination along the -k//z direction, such that the incoming light hits the top face of helicoid. |

| 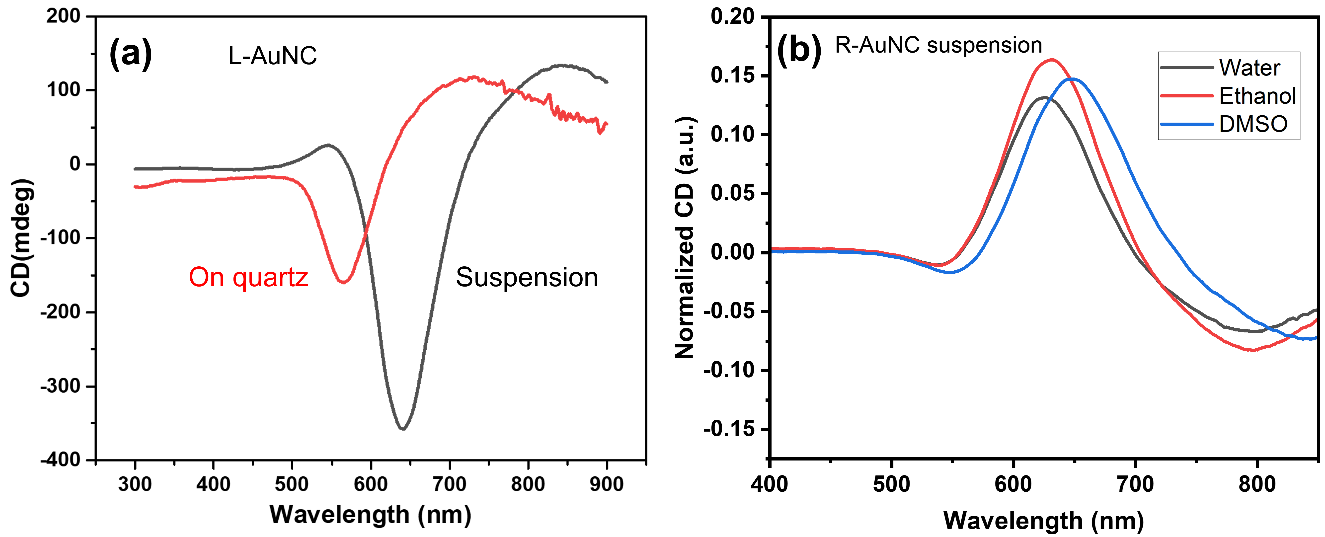 |
| --- |

**Figure S6.** (a) Comparison of circular dichroism (CD) spectra of L-AuNCs in solution and on a quartz substrate, showing a substrate-induced blue shift in the CD signal. (b) CD spectra of R-AuNCs dispersed in different media, illustrating a red shift in the CD peak with increasing refractive index of the surrounding environment.

| 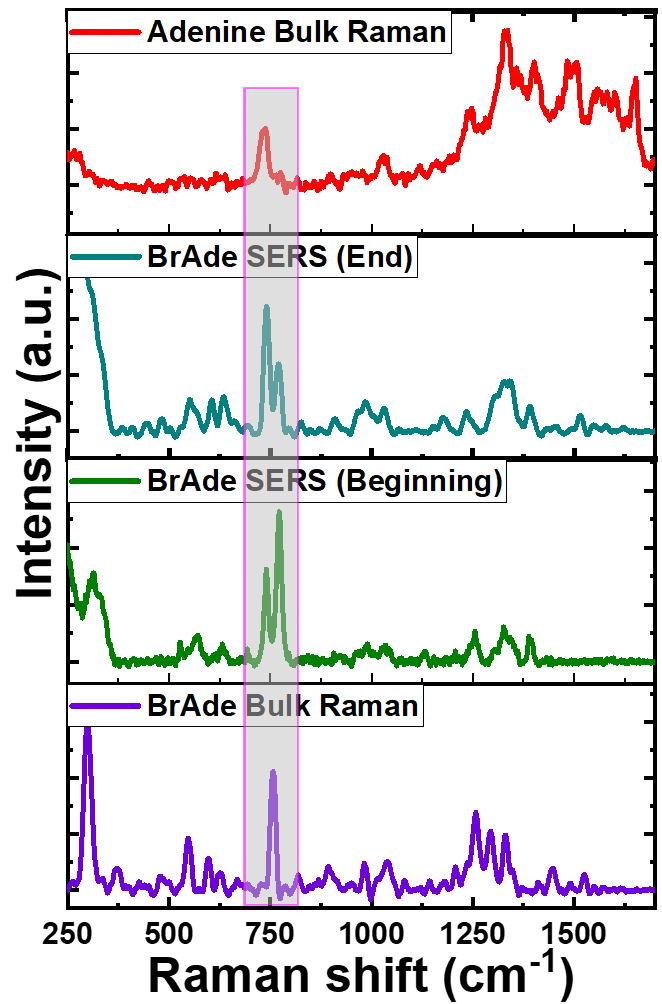 |
| --- |
| **Figure S7.** Bulk Raman spectra of the reactant and product (bottom and top), compared with the SERS spectrum of BrAde adsorbed on chiral AuNC particles (middle). The spectral region of interest (ring breathing modes) is highlighted for comparison. |

|  |
| --- |
| **Figure S8.** SERS spectra of 1 mM adenine solution using aggregated spherical AuNPs (60 nm diameter). The characteristic ring breathing mode of adenine appears at 740 cm⁻¹. |

| 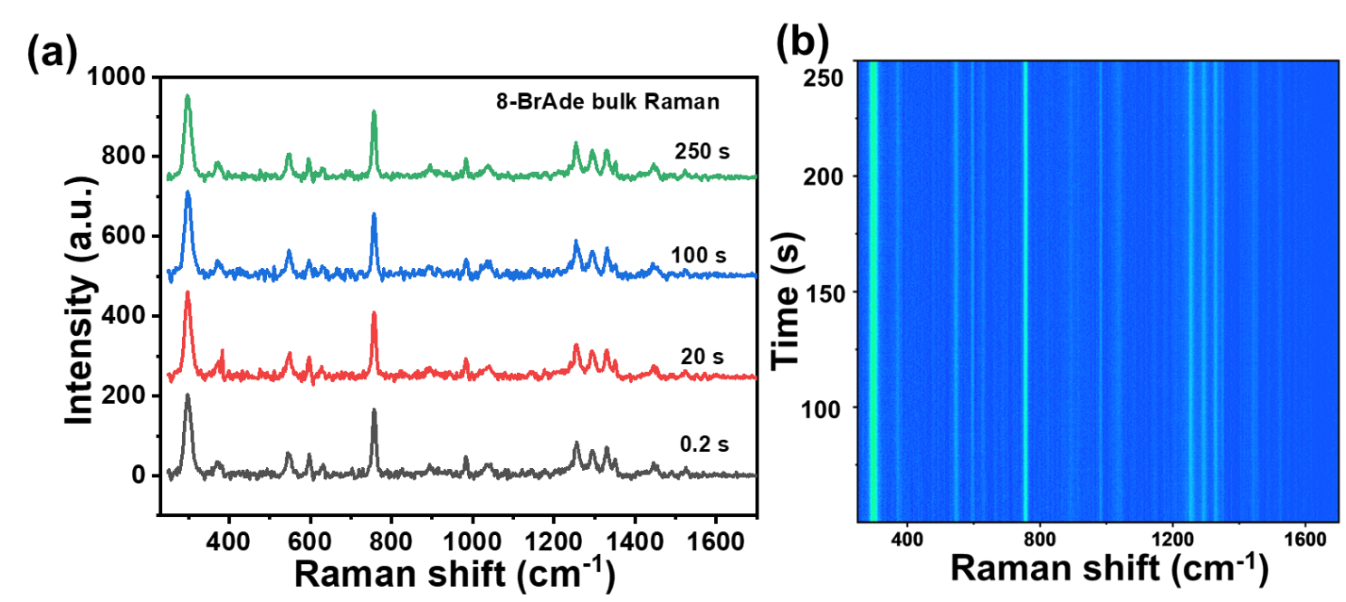 |
| --- |
| **Figure** **S9.** (a) Time-resolved Raman spectra of 8-bromoadenine (8-BrAde) powder under continuous illumination. (b) Corresponding contour plot showing no spectral changes over time, indicating that the reaction does not proceed in the absence of plasmonic nanoparticles. A 633 nm laser with 0.5 mW power was used for this measurement. |

| 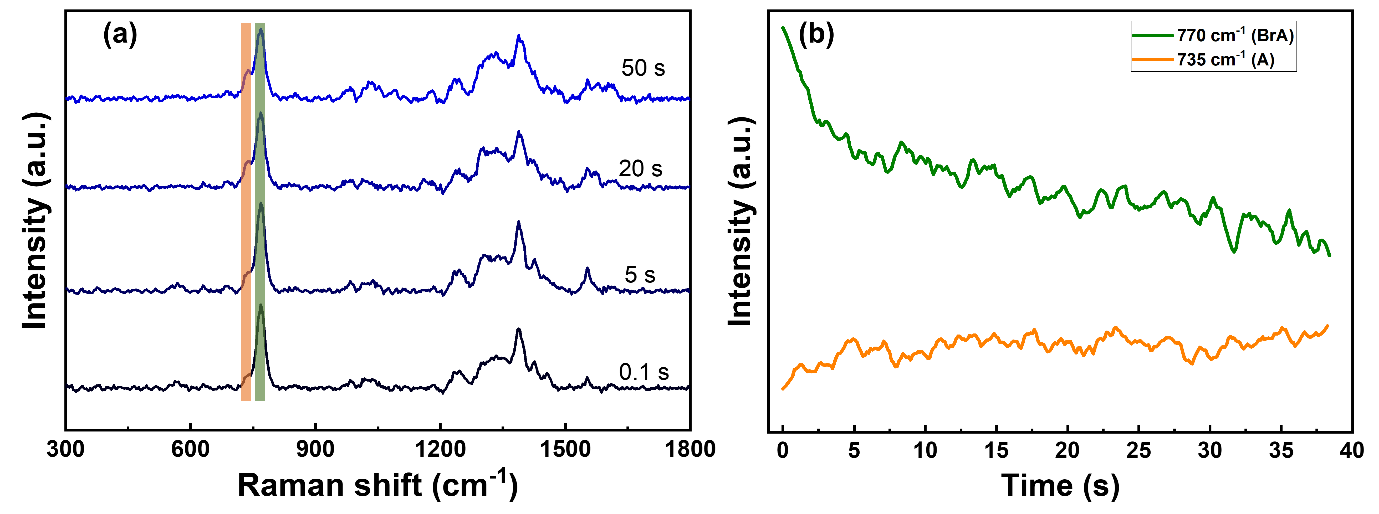 |
| --- |
| **Figure S10.** Time-series SERS measurements using achiral Au nanospheres (60 nm diameter). (a) Temporal evolution of SERS spectra of 8-bromoadenine (BrA), with key spectral changes highlighted. (b) Kinetics of the ring breathing mode intensities for BrA (770 cm⁻¹) and the product adenine (A, 735 cm⁻¹). Measurements were performed under 633 nm laser excitation at 0.5 mW using a 50× objective. The reaction proceeds slowly enough to capture an initial spectrum representative of the unreacted species. |

| **Rate constant ×10^-2^(s^-1^)** | **L-AuNC** | **R-AuNC** |
| --- | --- | --- |
| ***k*_LCP_** | **4.11 ± 0.45** | **5.49 ± 0.15** |
| ***k*_RCP_** | **5.78 ± 0.38** | **3.95 ± 0.14** |
| ***k*_Avg_=(*k*_LCP_ + *k*_RCP_)/2** | **4.94 ± 0.30** | **4.72 ± 0.12** |
| ***k*_LP_** | **4.50 ± 0.46** | **5.20 ± 0.14** |

**Table S1.** Values of rate constants of plasmon driven dehalogenation reaction with L-and R-AuNC under linearly and circulalry polarized illumination.

| 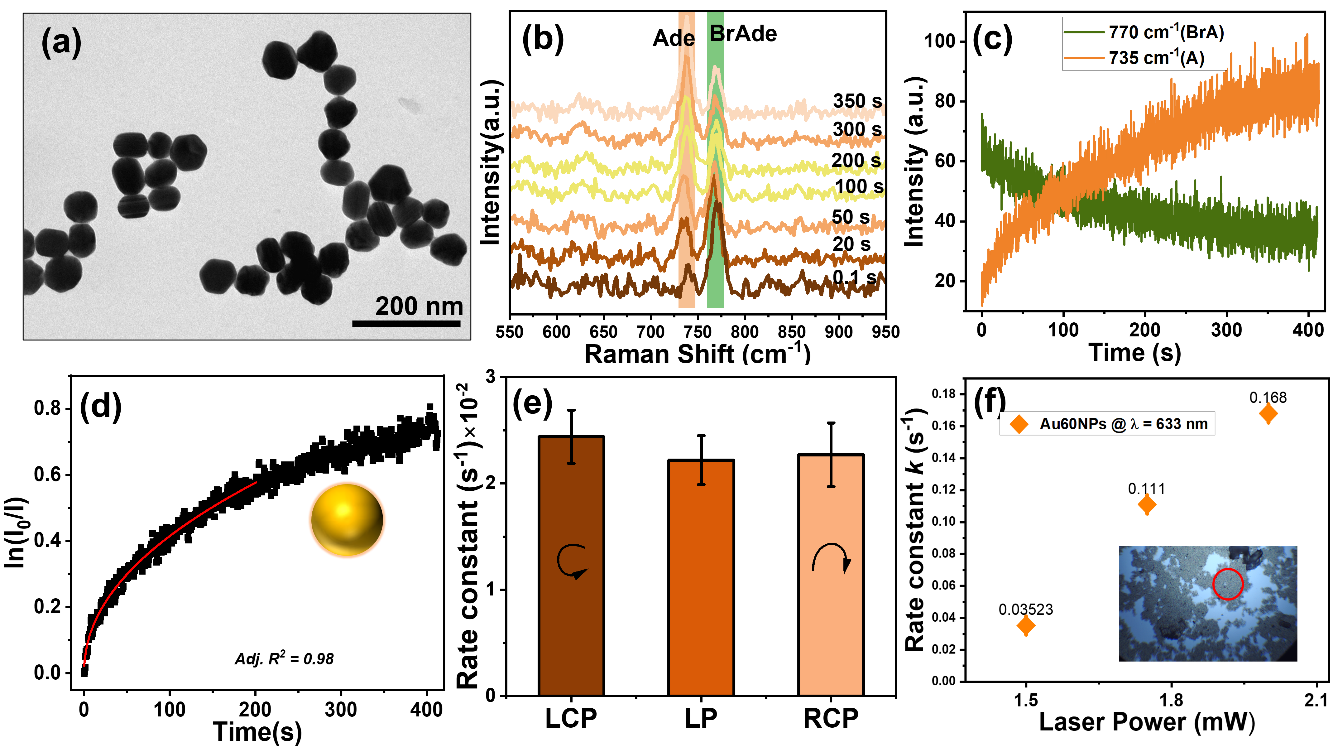 |
| --- |
| **Figure S11.** Control experiments using achiral Au nanospheres (60 nm diameter). (a) TEM image of the Au nanospheres. (b) Time-resolved SERS spectra of 8-bromoadenine (BrA). (c) Kinetics of peak intensities for the reactant (BrA, 770 cm⁻¹) and product adenine (A, 735 cm⁻¹). (d) Non-linear fit of the decay curve for rate constant extraction using Equation 12. (e) Bar graph comparing dehalogenation rate constants under linearly and circularly polarized light. (f) Dependence of the rate constant on laser power. Inset: optical image of the sample with the measurement area marked by a red circle. |

| 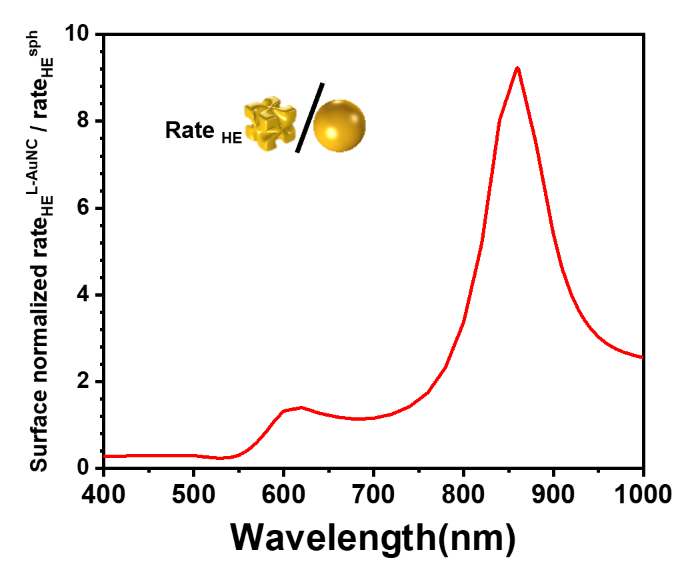 |
| --- |
| **Figure S12.** Simulated ratio of surface normalized rate of hot electron generation on single chiral L-AuNC and spherical AuNP (60 nm diameter). |

| 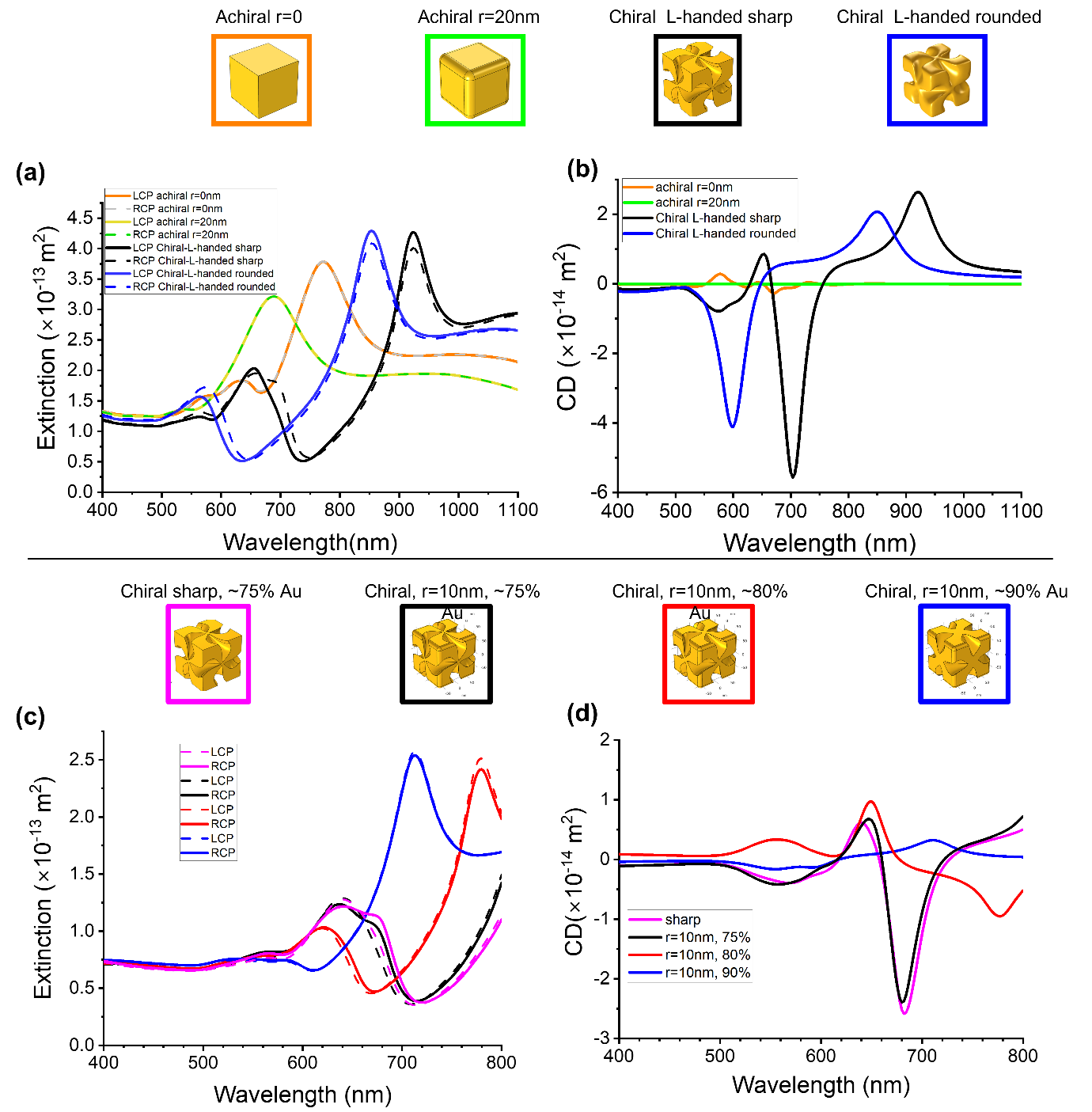 |
| --- |
| **Figure S13**. Differences in the optical response of perfect-cubes vs chiral cubes: (a,b) for a ~190 nm cube; and (c,d) for ~150 nm cube, for different cubic morphologies as shown in the schematics above each panel (r: edge rounding). In panels (c,d), and Au% refers to the amount of Au compared to the perfect achiral cubic case (100% gold), meaning that the less Au% the more pronounced the chiral corners, hence the more geometrically-chiral the cuboid). Panels (a) and (c) show extinction optical responses, and panels (b) and (d) show CD of extinctions. All simulations were carried out for helicoids in a water matrix, and an incoming light intensity of 3×10^7^ W/cm^2^ and the CPL illumination along the -k//z direction, such that the incoming light hits a face of helicoid. |

| 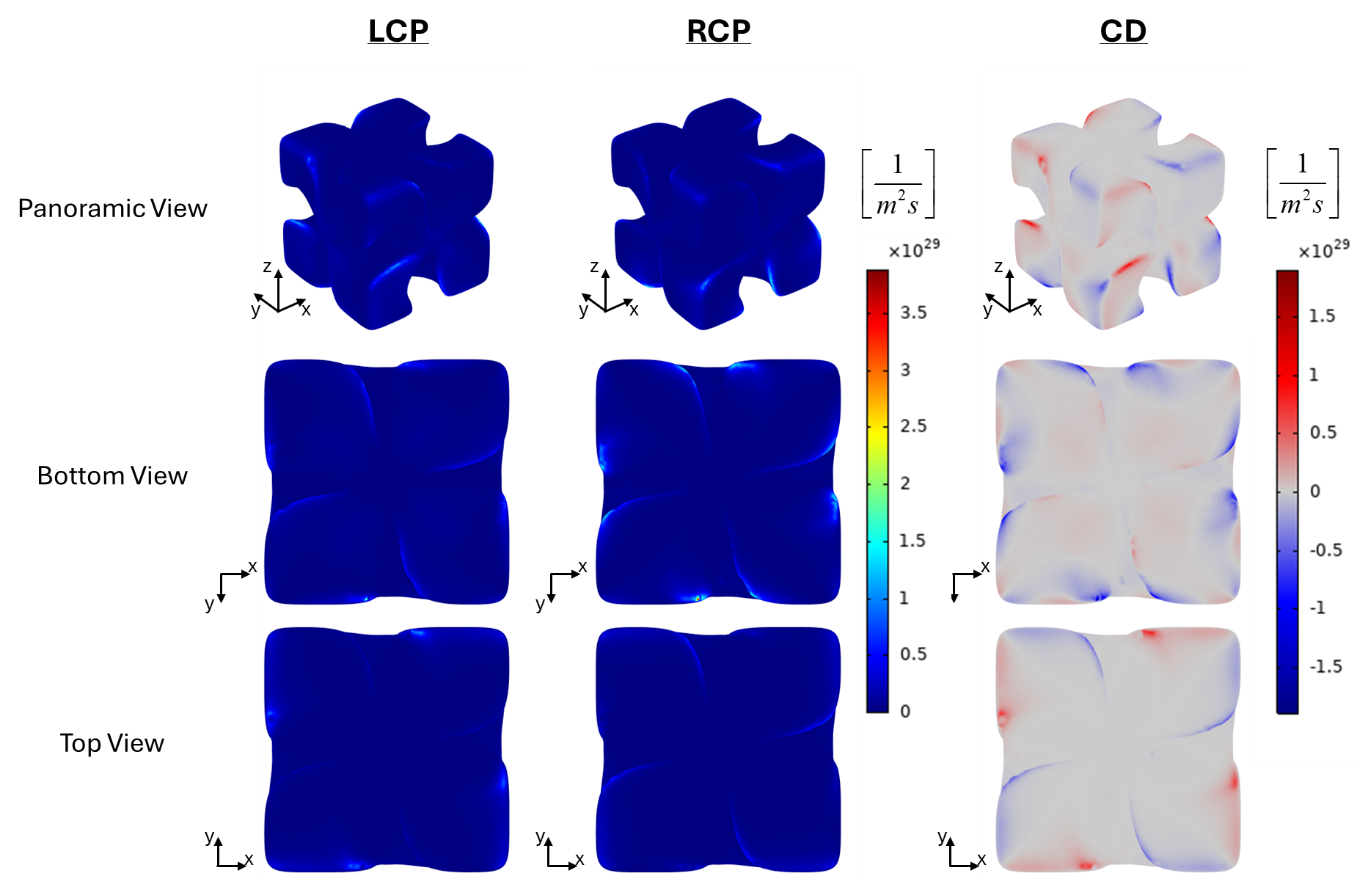 |
| --- |
| **Figure S14.** Simulated hot electron generation and CD maps on single L-AuNC in solution, illuminated with LCP and RCP at **520 nm** calculated by COMSOL. The simulated cubes are 190 nm on the edge, illumination is from above orthogonally into the face, k//-z. |

| 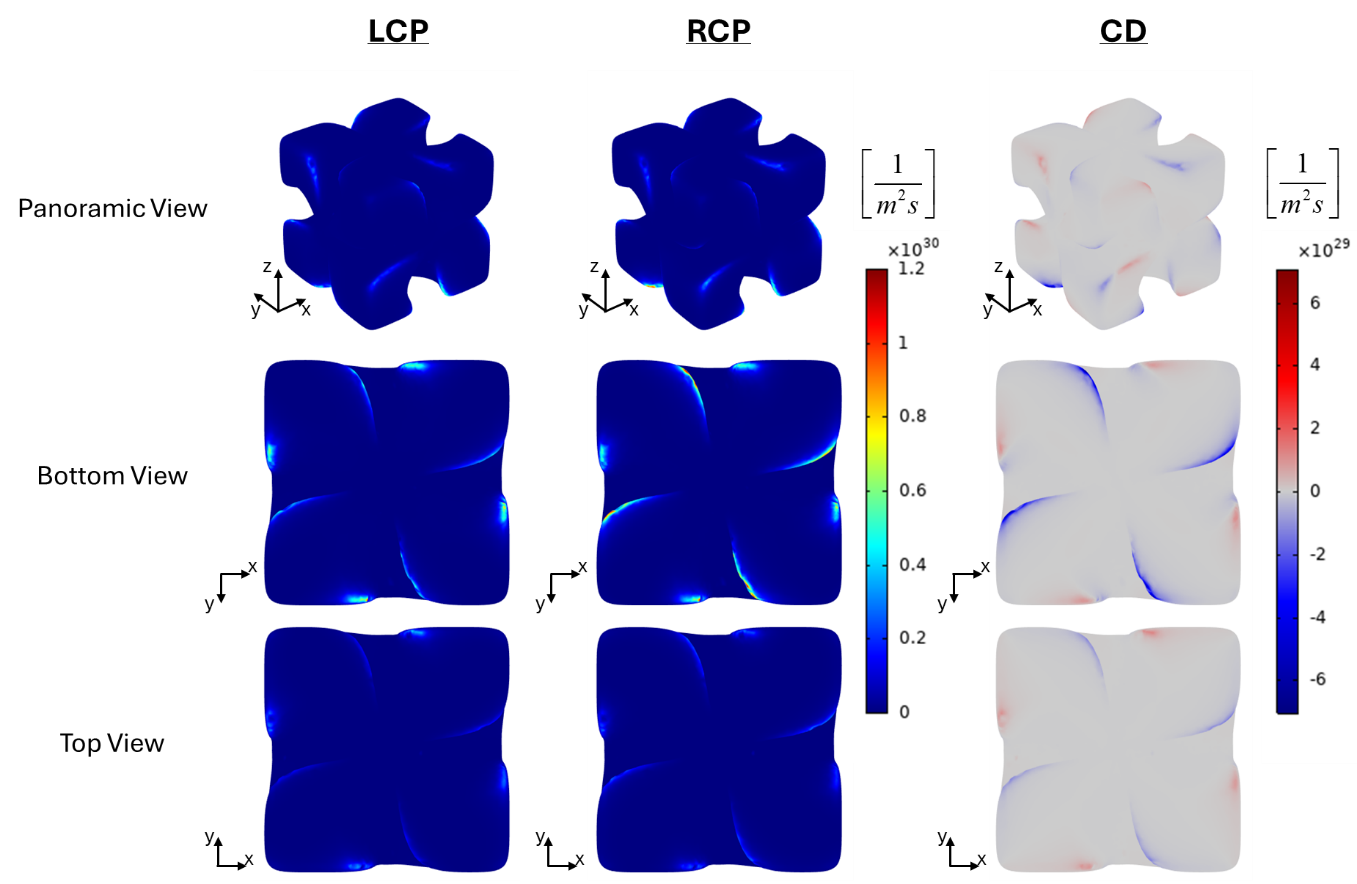 |
| --- |
| **Figure S15.** Simulated hot electron generation and CD maps on single L-AuNC in solution, illuminated with LCP and RCP at **599 nm** excitation calculated by COMSOL. The simulated cubes are 190 nm on the edge, illumination is from above orthogonally into the face, k//-z. |
| 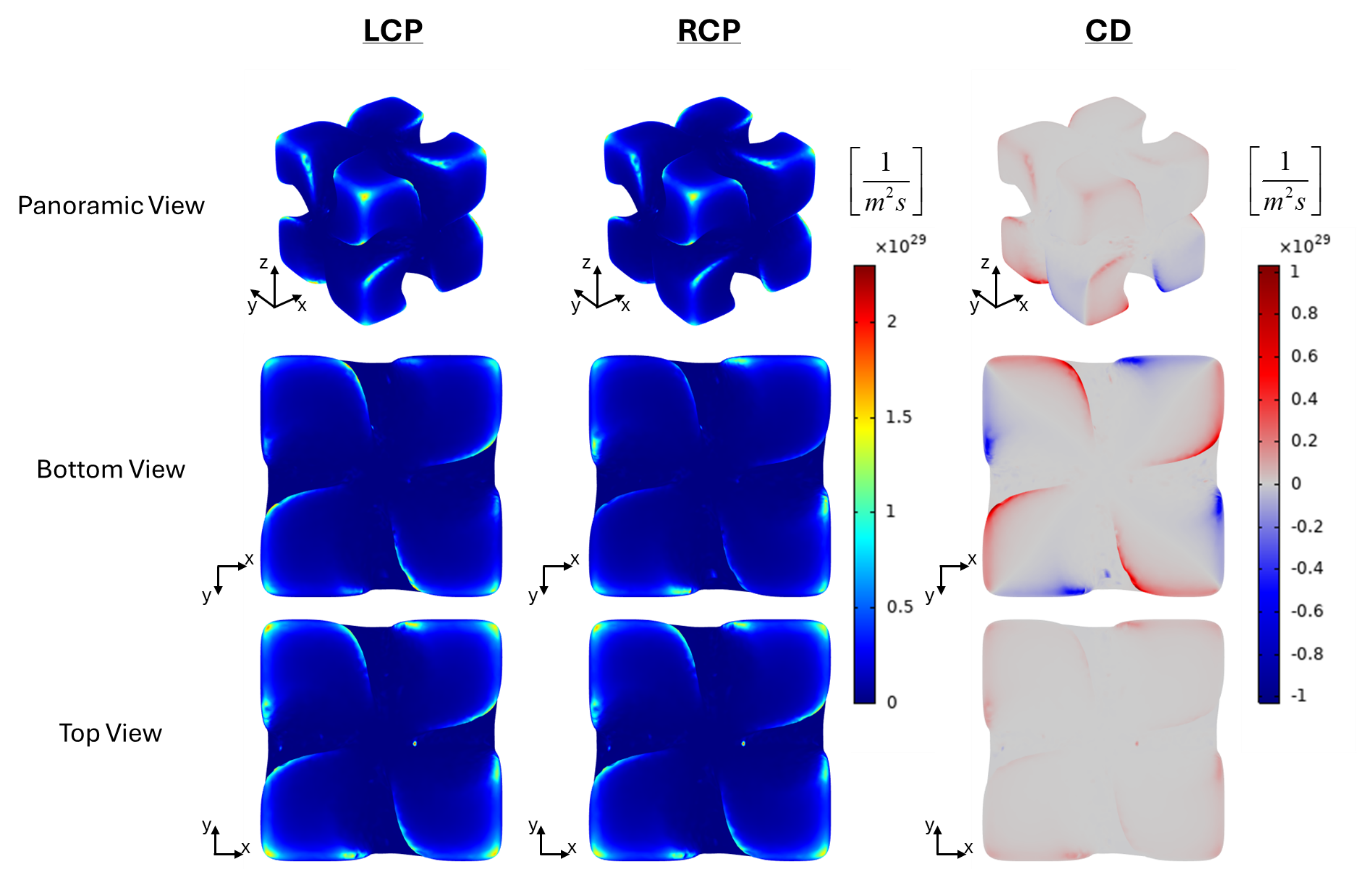 |
| **Figure S16.** Simulated hot electron generation and CD maps on single L-AuNC in solution, illuminated with LCP and RCP at **850 nm** excitation calculated by COMSOL. The simulated cubes are 190 nm on the edge, illumination is from above orthogonally into the face, k//-z. |

| 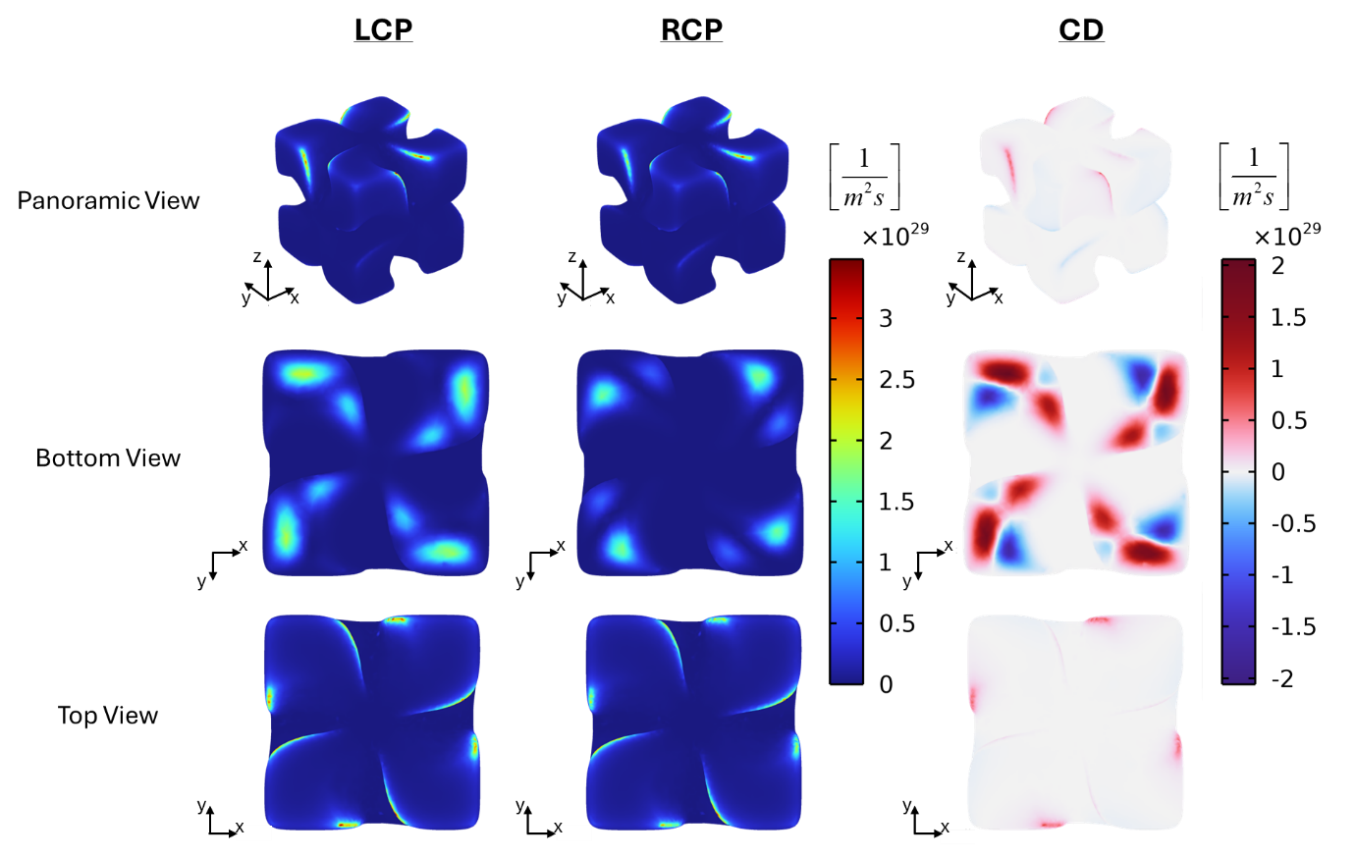 |
| --- |
| **Figure S17.** Simulated hot electron generation and CD maps on single L-AuNC on a Si-substrate, illuminated with LCP and RCP at **599 nm** excitation calculated by COMSOL. The simulated cubes are 190 nm on the edge, illumination is from above orthogonally into the face, k//-z. |

| 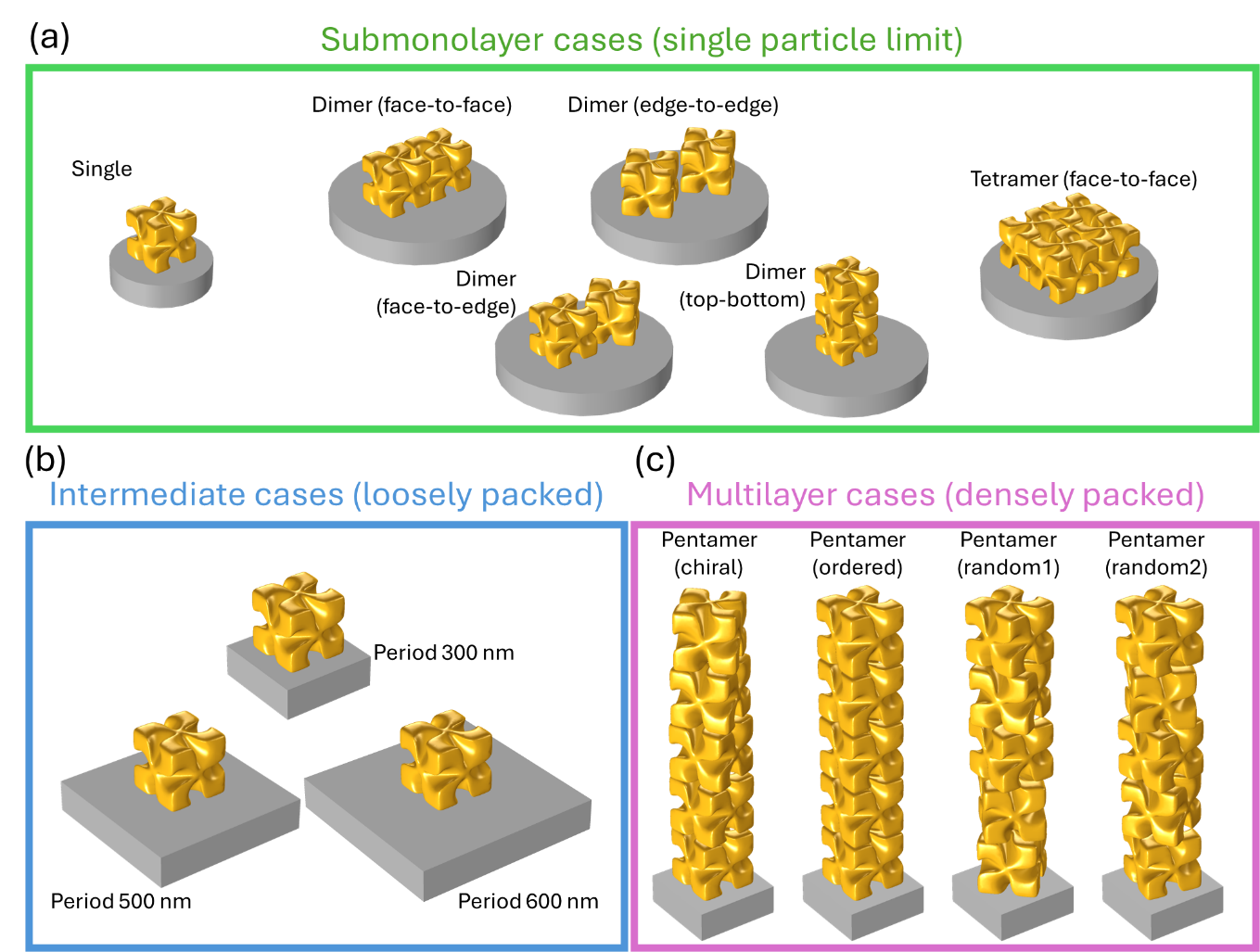 |
| --- |
| **Figure S18.** Three simulated representative HE-generation regimes: (a) single-particle limit, (b) loosely packed, and (c) densely packed, as shown. Within each regime, several possible systems are considered. The HE generation rates of each system are calculated for a normal-incidence (k//-z) CPL excitation of 633 nm, after which values are averaged and shown as a single data point in Figure S22. (a) In the single-particle limit regime, each shown system is individually set on an infinite silicon substrate, without other NPs present at all. (b) In the loosely packed regime, each system shown is a unit cell of a periodic infinite system, with period as shown. (c) In the densely packed regime, each of the four systems is a unit cell of a periodic infinite system, with period $200\sqrt{2}$ nm. In all systems $I_{0}=3\times{10}^{7}\left[ \text{W/}\text{cm}^{\text{2}} \right].$ |

| 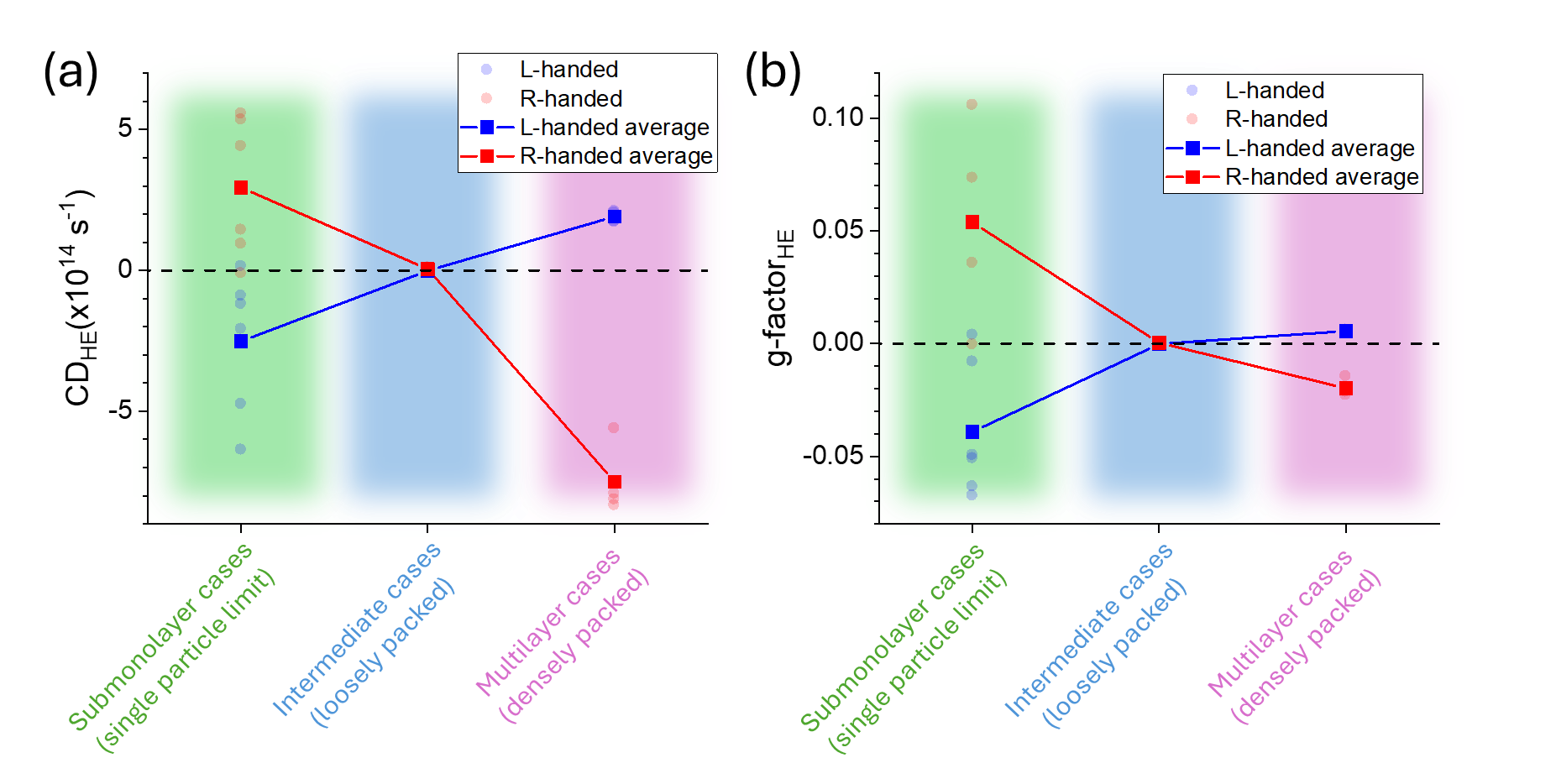 |
| --- |
| **Figure S19.** Simulated HE-generation CD and g-factor for the three simulated regimes: single-particle limit (green region), loosely packed (blue region), and densely packed (purple region). Each regime is schematically described in Figure S21. The simulations are carried out for several systems of L-handed AuNC (blue data) and R-handed AuNC (red data) under same conditions. Each system’s numerical data is shown as a light circle symbol, whereas the average over the entire regime is shown as a solid square symbol. One can see the change in chirality when going from the single-particle limit to densely–packed cases. In all systems $I_{0}=3\times{10}^{7}\left[ \text{W/}\text{cm}^{\text{2}} \right].$ |

| 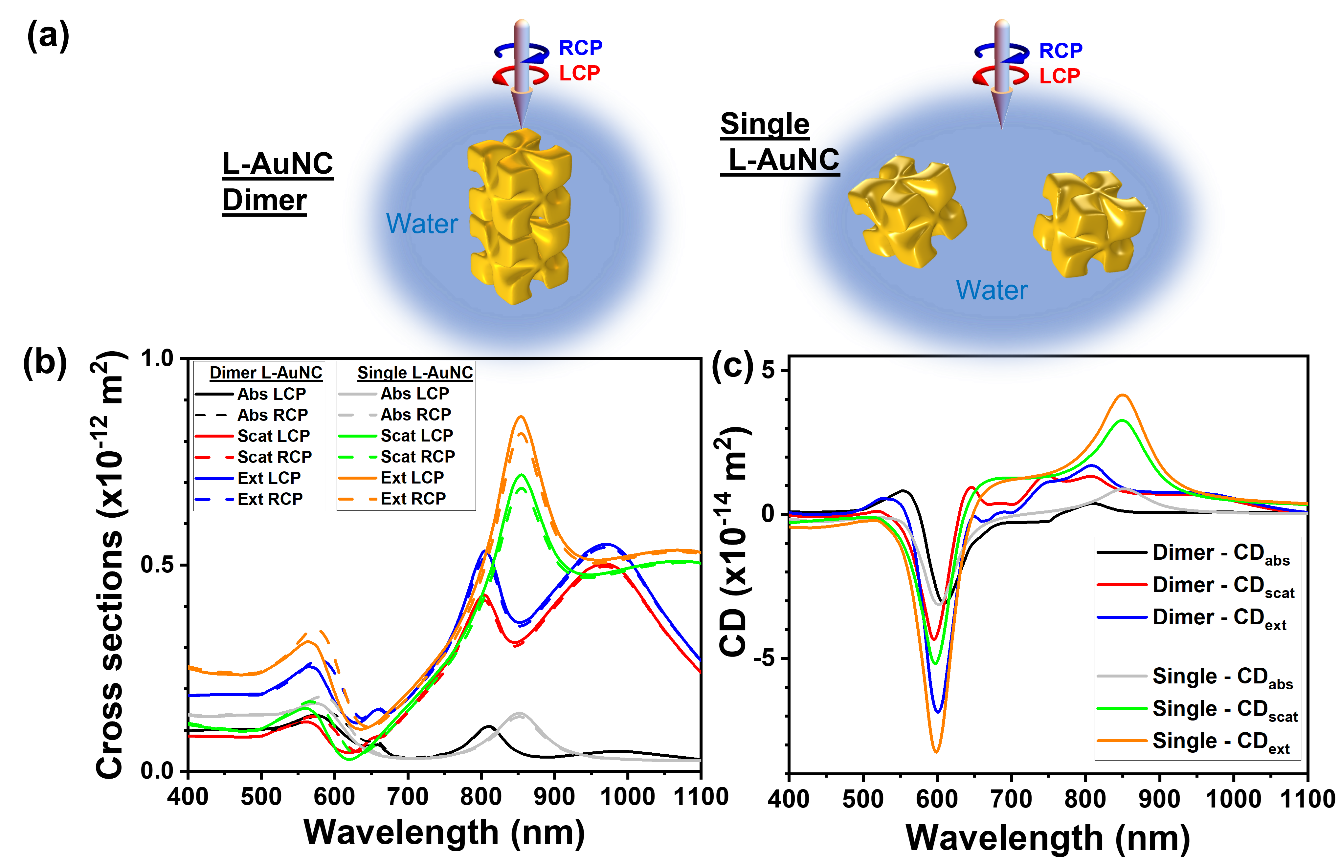 |
| --- |
| **Figure S20.** (a) Schematic showing the CPL illumination on vertical dimers and single L-AuNCs dispersed in water (b) Absorbance, scattering and extinction and (c) CD spectra in both orientations at different handedness of CPL. |

| 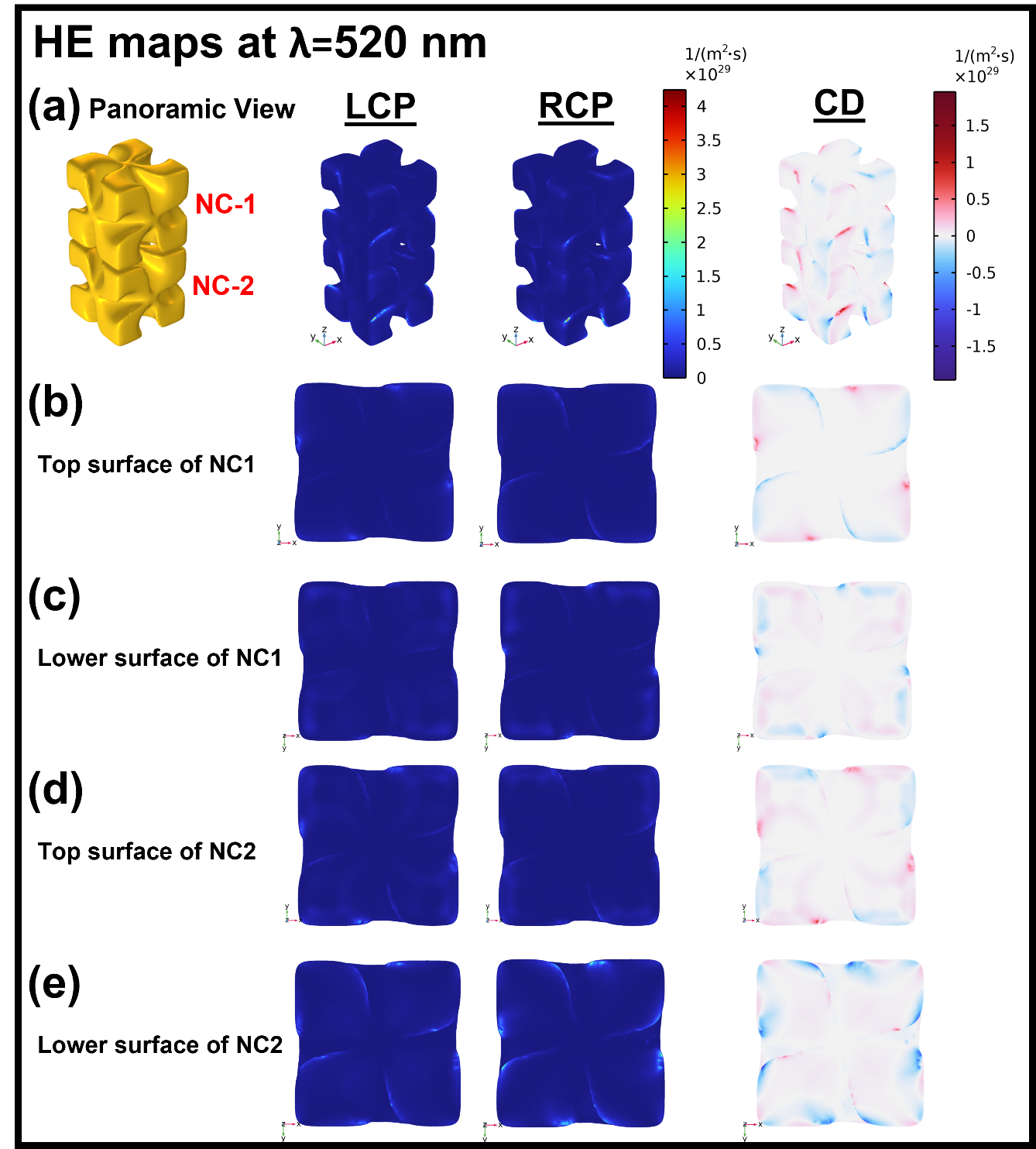 |
| --- |
| **Figure S21.** Hot electron (HE) maps and CD on vertical L-AuNCs dimer in water illuminated with LCP and RCP at 520 nm excitation calculated by COMSOL. Direction of illumination k\|\|-z. |

| 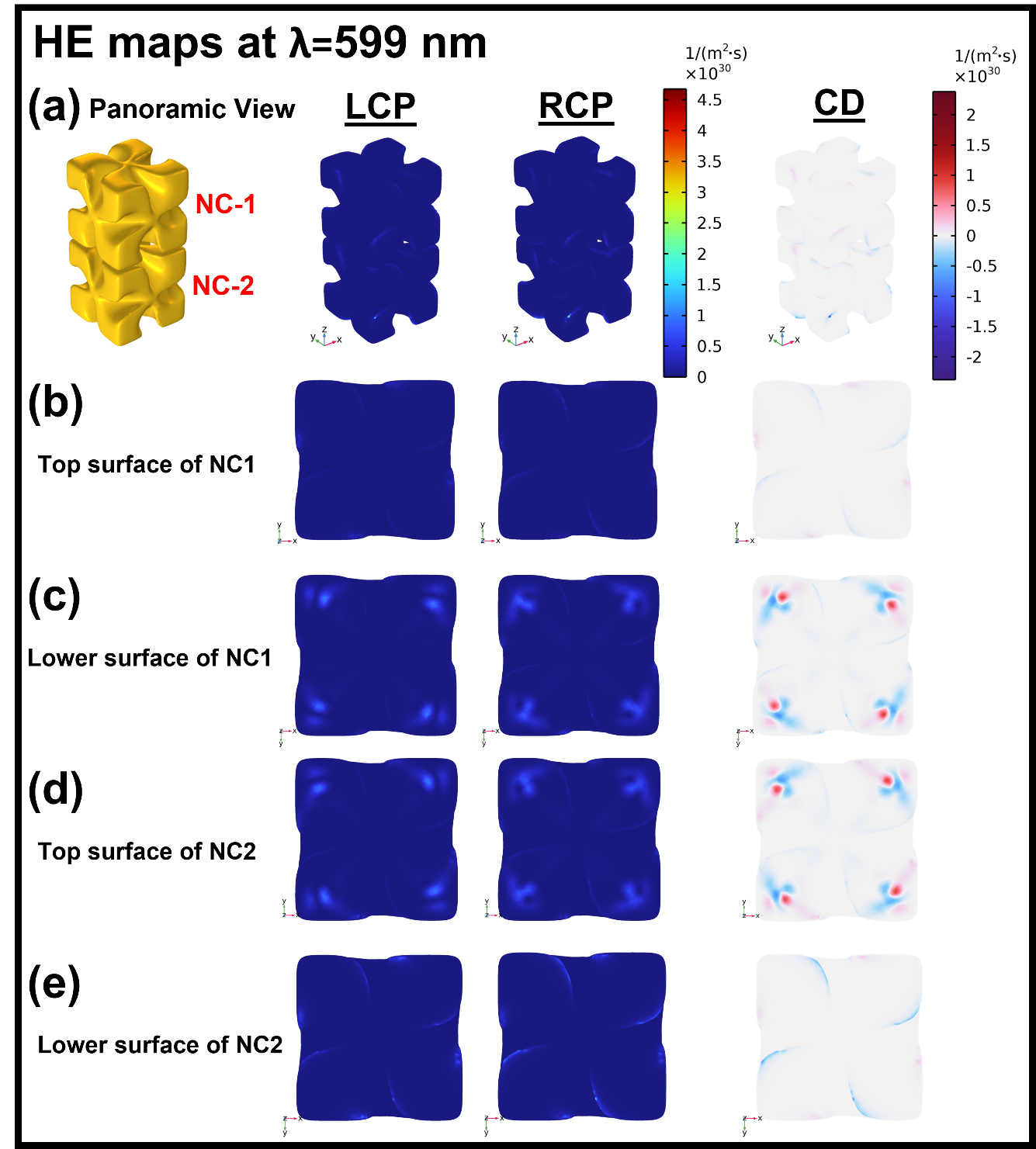 |
| --- |
| **Figure S22.** Hot electron (HE) maps and CD on vertical L-AuNCs dimer in water illuminated with LCP and RCP at **599 nm** excitation calculated by COMSOL. Direction of llumination: k\|\|-z. Positive values of CD are observed on the strong hot-spots where the AuNCs are facing each other, shown in (C) and (D). Which can support the experimental results of reversed CD in case of stacking of AuNCs. |

| 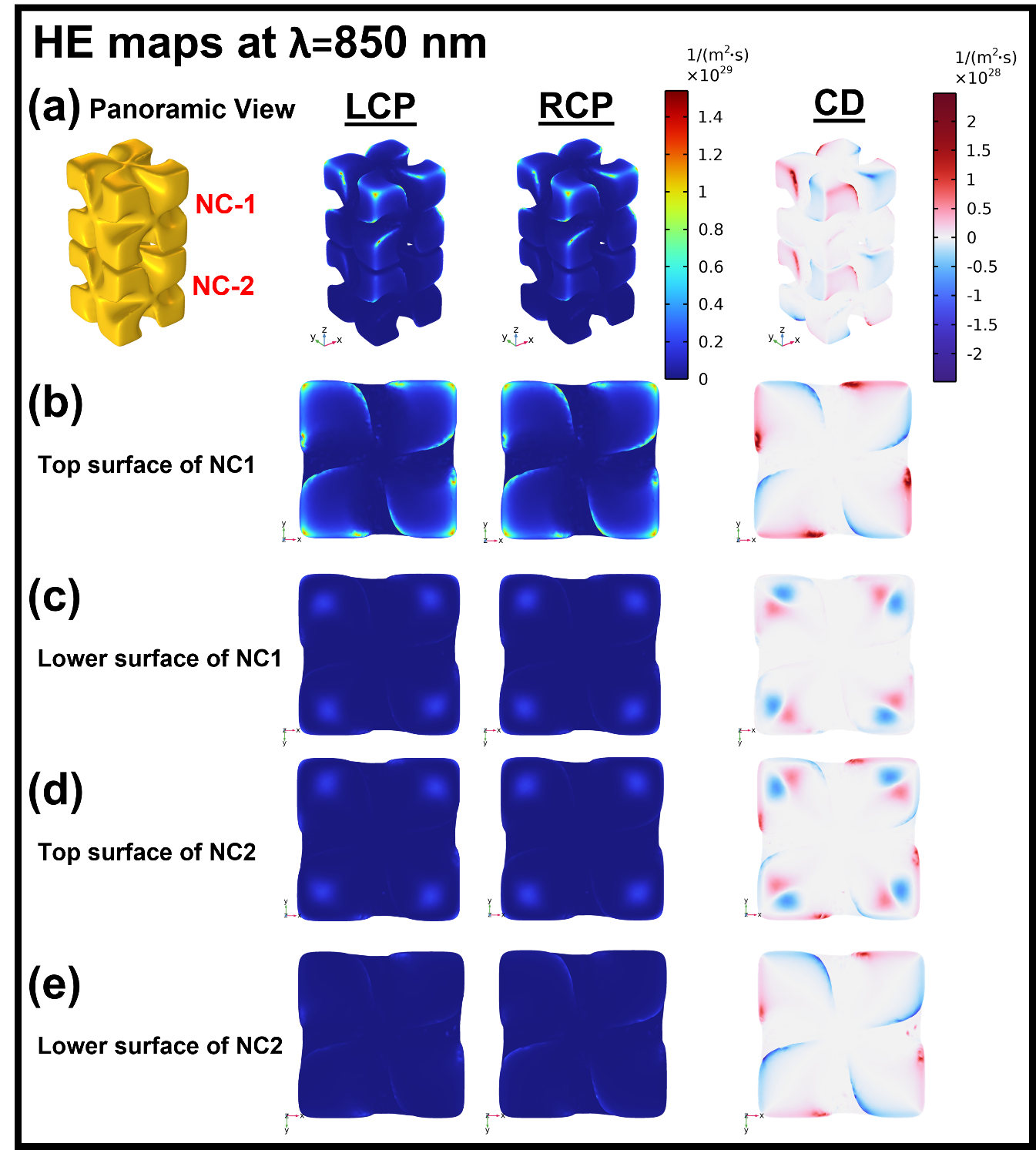 |
| --- |
| **Figure S23.** Hot electron (HE) maps and CD on vertical L-AuNCs dimer in water illuminated with LCP and RCP at 850 nm excitation calculated by COMSOL. Direction of llumination: k\|\|-z. |

| 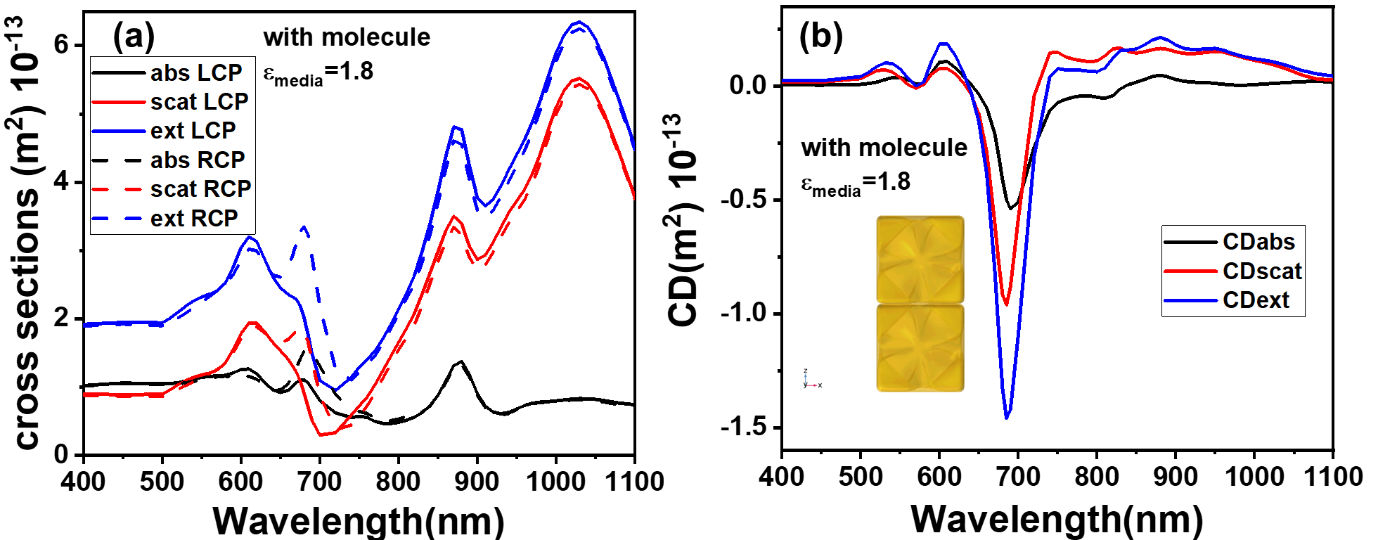 |
| --- |
| **Figure S24.** Theoretical calculations of the optical properties of a vertical dimer composed of L-AuNCs with an adsorbate, in a surrounding medium of permittivity 1.8. (a) Absorption, scattering, and extinction cross-sections under left- and right-circularly polarized (LCP and RCP) illumination. (b) Corresponding optical circular dichroism (CD) spectrum of the same dimer system. |

| 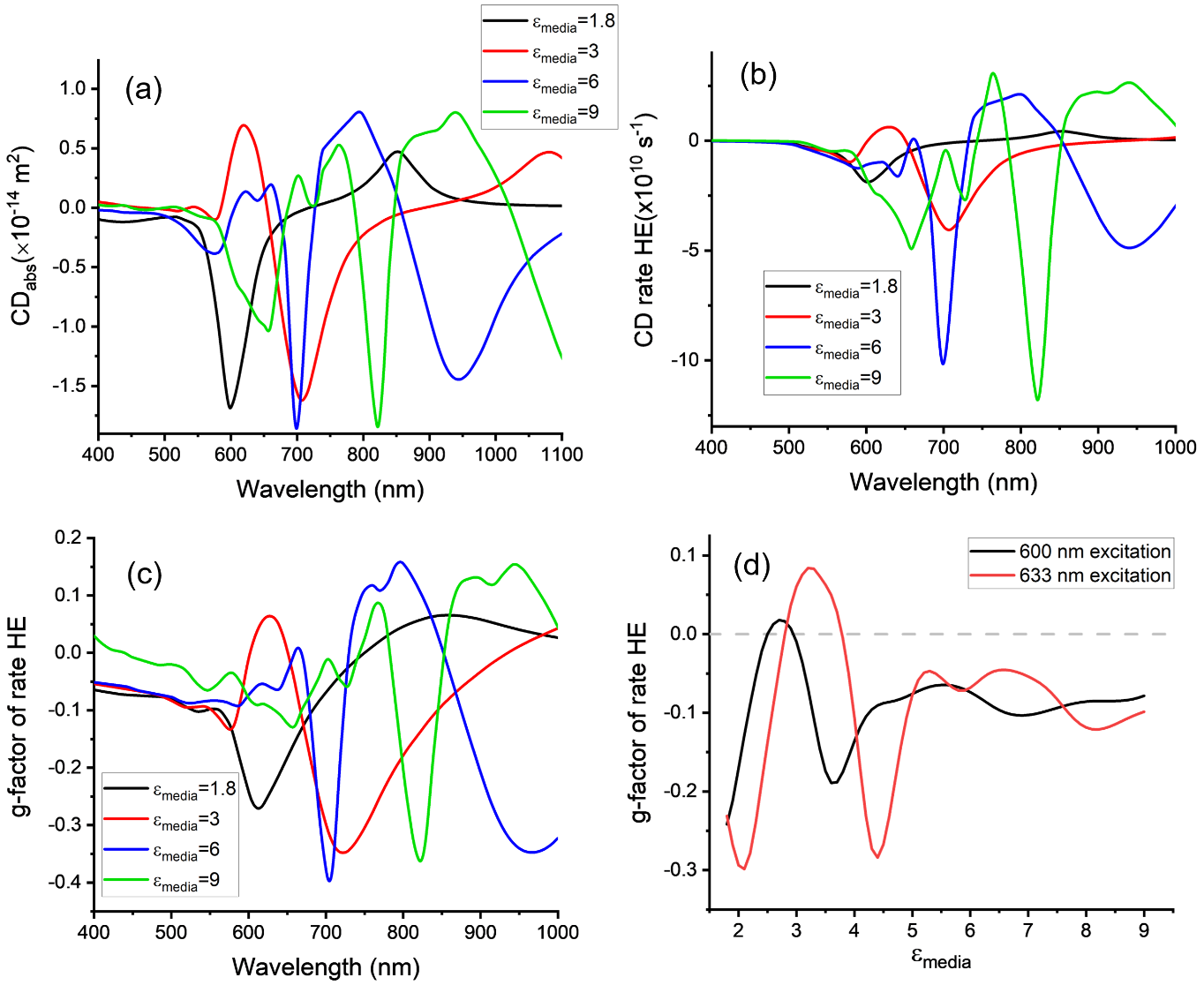 |
| --- |
| **Figure S25.** Simulated effects of varying effective media permittivity. (a, b) Absorption and hot electron generation rate circular dichroism (CD) spectra as functions of excitation wavelength for four different permittivity values. (c) g-factors of the hot electron generation rate at these permittivity values. (d) g-factors as a function of permittivity for two selected excitation wavelengths. (e) CD spectra of hot electron generation rates corresponding to panel (b). All simulations were performed at $I_{0}=400\left[ \text{W/}\text{cm}^{\text{2}} \right].$ |

| 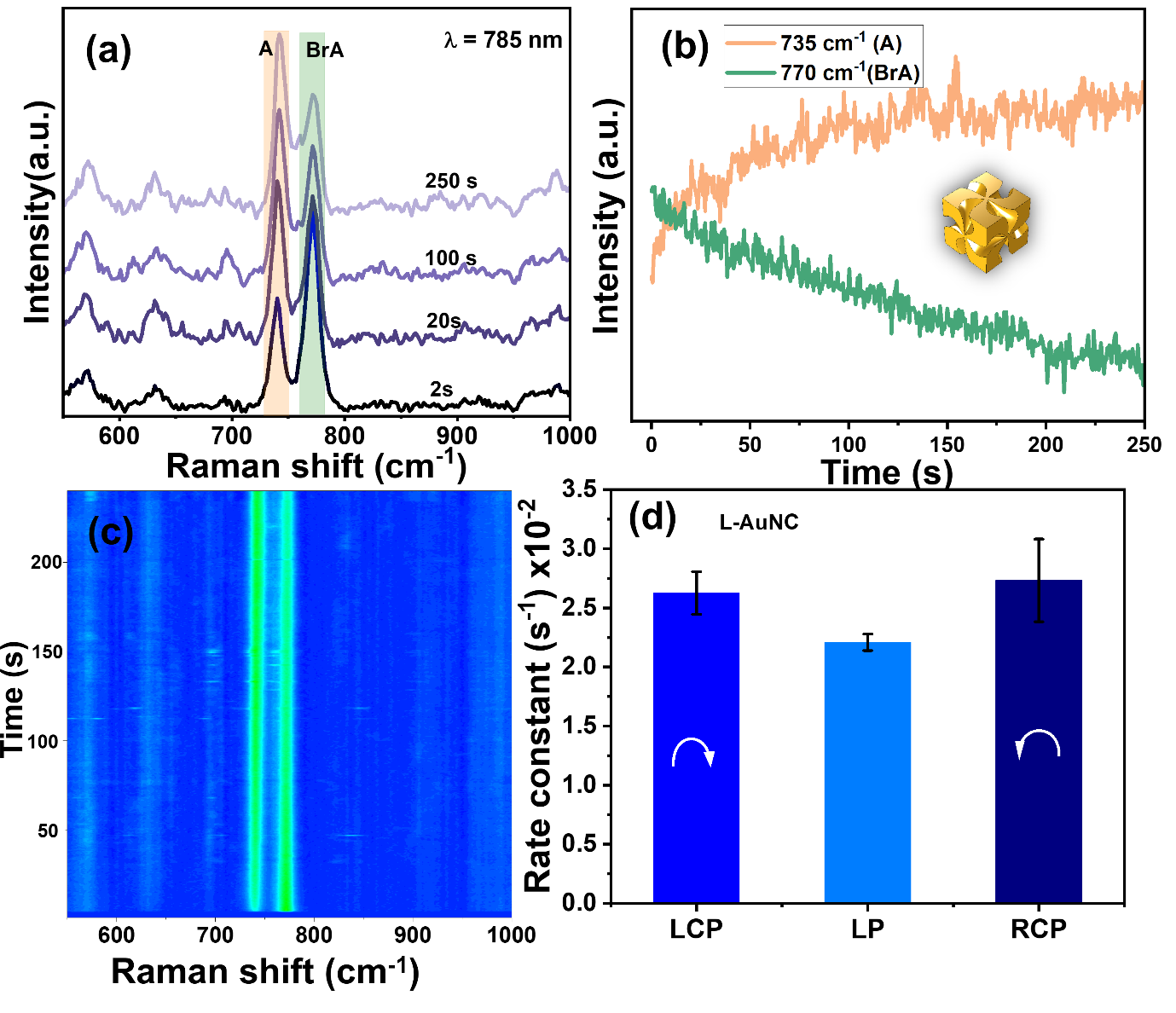 |
| --- |
| **Figure S26.** Plasmon-induced transformation of 8-bromoadenine (BrA) into adenine (A) using a 785 nm laser on L-AuNCs. (a) Time-resolved SERS spectra of BrA, with key spectral changes highlighted. (b) Kinetics of the ring breathing mode intensities for BrA (770 cm⁻¹) and A (735 cm⁻¹). (c) Contour plot showing SERS signal evolution over time. (d) Reaction rate constants extracted under different polarization states of the incident light. |
|  |
| **Figure S27.** Laser power dependence of rate constant and g-factor at 785 nm laser excitation with R-AuNC deposited on Si. |

| 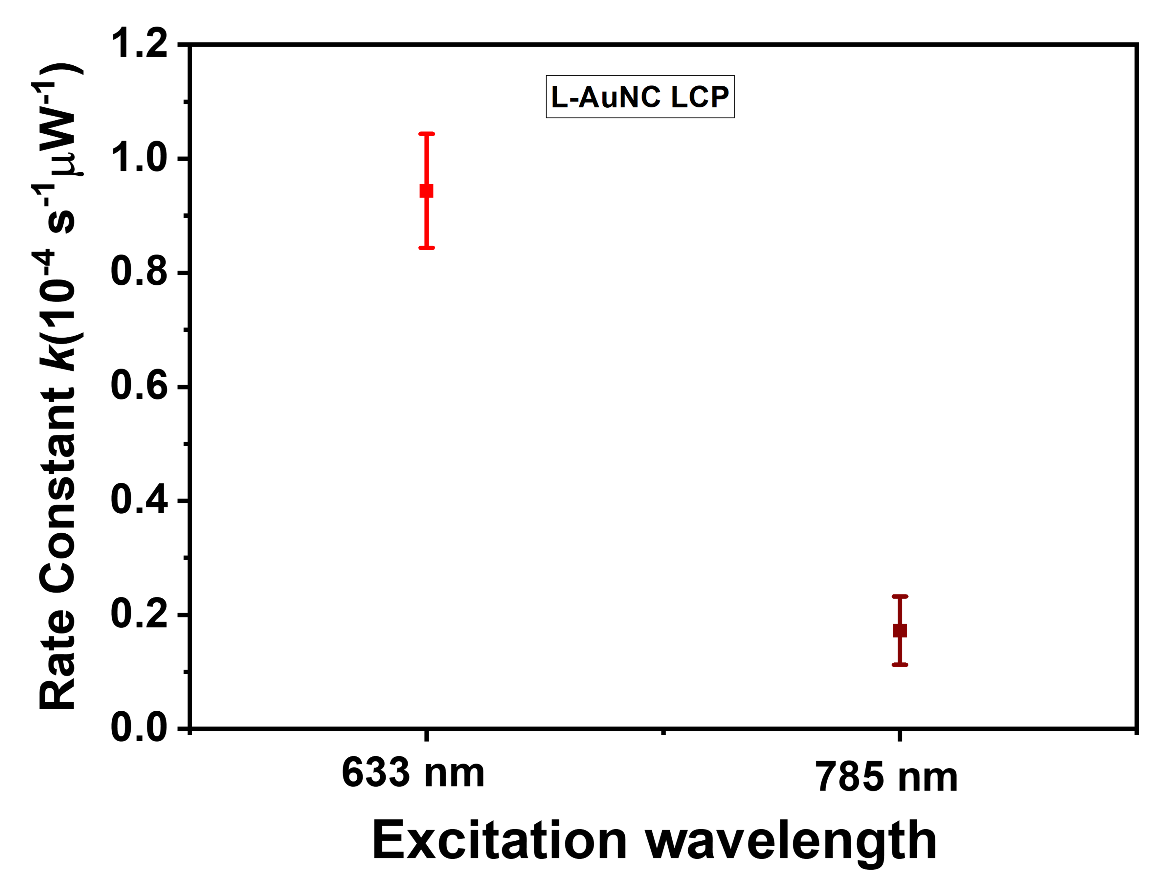 |
| --- |
| **Figure S28.** Excitation wavelength dependence of rate constants with L-AuNC irradiated with LCP at two different laser excitations. |

| 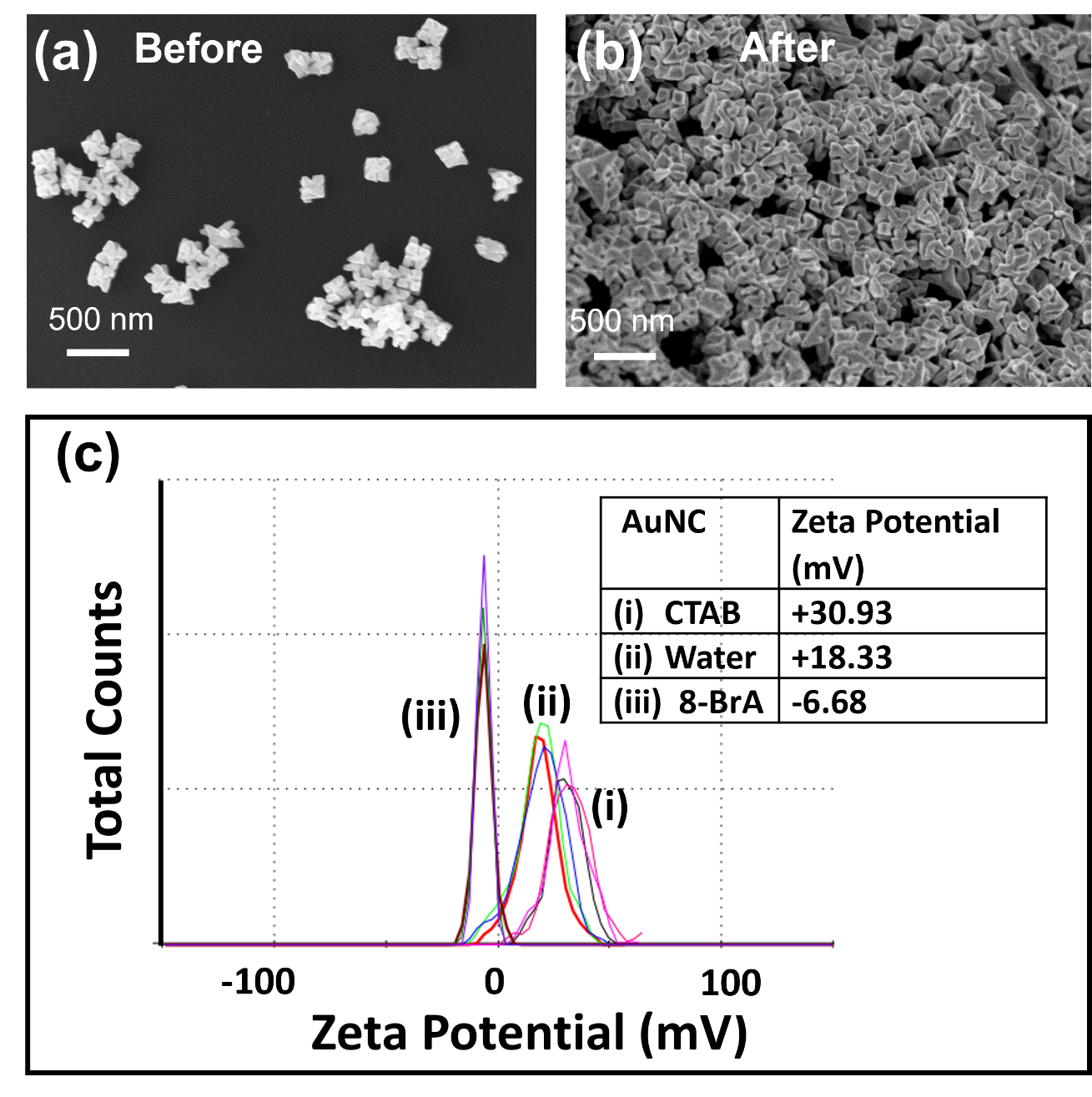 |
| --- |
| **Figure S29.** SEM and zeta potential analysis demonstrating the structural and surface stability of L-AuNCs under laser exposure. (a) SEM image of pristine, unfunctionalized nanocubes. (b) SEM image after time-series SERS measurements with 8-bromoadenine (BrA) coating. (c) Zeta potential measurements (three replicates) before and after BrA functionalization. The observed shift in zeta potential confirms surfactant displacement by the reactant, indicating successful surface modification. |

| 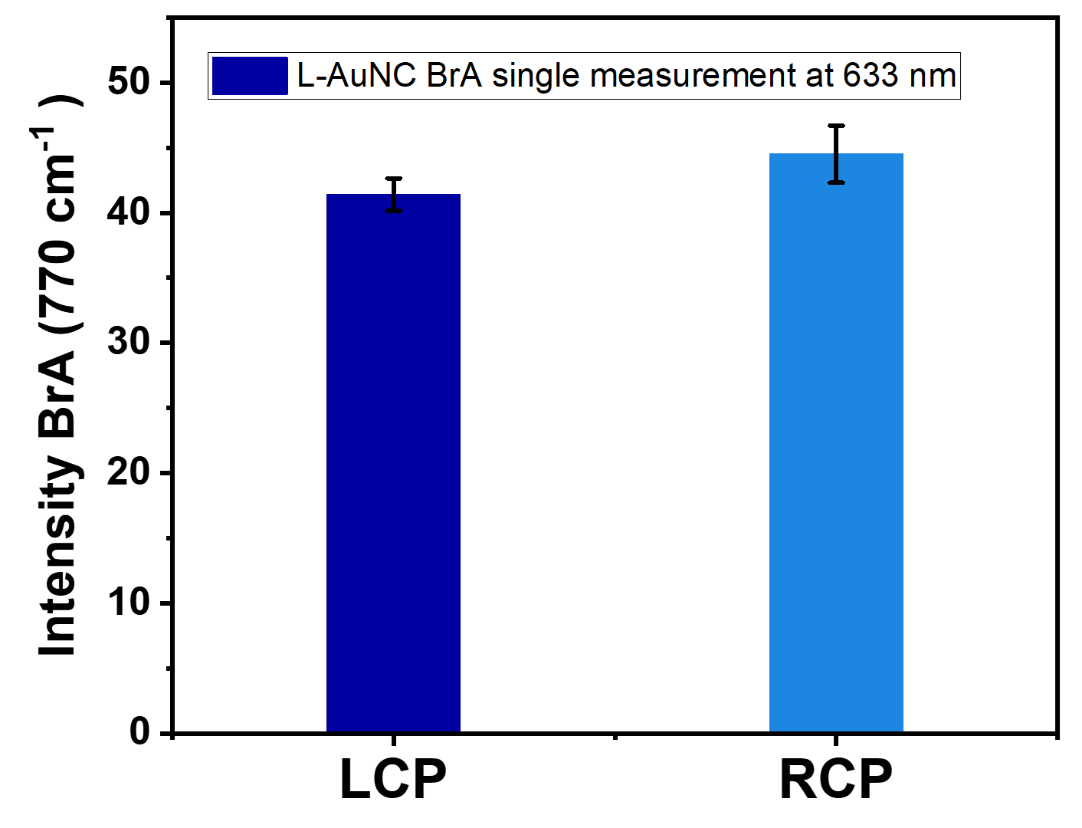 |
| --- |
| **Figure S30.** Surface-enhanced Raman intensity comparison of the ring breathing mode of 8-bromoadenine (BrA) at the initial time point under left- (LCP) and right-circularly polarized (RCP) light illumination on L-AuNCs. |
